# Metabolism in Tumor: Mechanisms and Therapeutic Perspectives

**DOI:** 10.1002/mco2.70928

**Published:** 2026-08-26

**Authors:** Jia‐Chen Cheng, Yi‐Xuan Hu, Xiang‐Chun Huang, Shan Jiang, Jie‐Lin Wang, Yang Zhao, Shuo Chen

**Affiliations:** ^1^ Department of Obstetrics and Gynecology Department of Gynecologic Oncology Research Office Guangzhou Medical University Guangzhou China; ^2^ Guangzhou Key Laboratory of Targeted Therapy for Gynecologic Oncology Guangzhou Medical University Guangzhou China; ^3^ Guangdong Provincial Key Laboratory of Major Obstetric Diseases Guangzhou Medical University Guangzhou China; ^4^ Guangdong Provincial Clinical Research Center for Obstetrics and Gynecology Guangzhou Medical University Guangzhou China; ^5^ Guangdong‐Hong Kong‐Macao Greater Bay Area Higher Education Joint Laboratory of Maternal‐Fetal Medicine Guangzhou Medical University Guangzhou China; ^6^ The Third Affiliated Hospital Guangzhou Medical University Guangzhou China; ^7^ Cancer Hospital of Dalian University of Technology Cancer Hospital of China Medical University Liaoning Cancer Hospital & Institute Shenyang China

**Keywords:** combination immunotherapy, drug resistance, HIF‐1α–glycolysis, histone lactylation, metabolic plasticity, nanomedicine, SREBP–FASN axis, tumor metabolism, tumor microenvironment

## Abstract

Tumor metabolic reprogramming now extends far beyond the Warburg effect, spanning glycolysis, mitochondrial oxidative phosphorylation (OXPHOS), de novo lipogenesis, cholesterol biosynthesis, and fatty acid oxidation. This network is shaped by cell‐intrinsic regulatory layers—transcriptional programs, histone lactylation and other posttranslational modifications, and m^6^A RNA modification—and by metabolic crosstalk with tumor‐associated macrophages (TAMs) and cancer‐associated fibroblasts (CAFs) that drives immune evasion and drug resistance. How these diverse inputs converge remains undefined: no integrated framework connects regulatory layers to signaling networks such as PI3K–AKT–mTOR, HIF‐1α, and cGAS–STING, or explains how their convergence generates metabolic plasticity. This review organizes tumor metabolic reprogramming into functional modules centered on three convergence hubs: the SREBP1/2–FASN–SCD1 lipogenic axis, the HIF‐1α–GLUT1–LDHA glycolytic axis, and the LDLR–SCARB1–LXR cholesterol homeostasis system. The discussion traces how multilayered regulation sustains each hub, how metabolic competition between tumor cells and immune effectors creates immunometabolic checkpoints, and how compensatory rewiring undermines single‐agent inhibitors. Therapeutic strategies span rational combination regimens that preempt compensatory rewiring to nanomedicine platforms enabling spatially controlled metabolism–immunity reprogramming. By identifying the hubs upon which diverse oncogenic signals converge, this framework reveals context‐specific metabolic vulnerabilities and outlines priorities for biomarker‐driven patient stratification and hub‐targeted combination therapy.

## Graphical abstract

Tumor metabolic reprogramming converges on three hubs—the SREBP1/2–FASN–SCD1 lipogenic axis, the HIF‐1α–GLUT1–LDHA glycolytic axis, and the LDLR–SCARB1–LXR cholesterol system—which integrate transcriptional, epigenetic (histone lactylation, m^6^A), and microenvironmental inputs to generate metabolic plasticity, immune evasion, and therapy resistance. Targeting these hubs, rather than individual enzymes, through combination and nanomedicine strategies enables biomarker‐driven, hub‐targeted therapy.

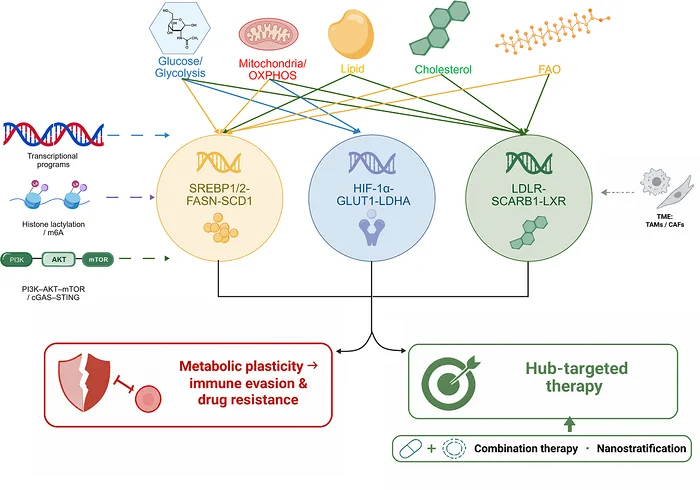

## 1 Introduction

Tumor metabolic reprogramming has been recognized as a hallmark of cancer since the discovery of aerobic glycolysis nearly a century ago [1]. The intervening decades have transformed this field from a glycolysis‐centric view into a multidimensional understanding in which cancer cells dynamically rewire glucose, lipid, cholesterol, and mitochondrial metabolism—not merely to generate ATP and biosynthetic precursors, but to sustain redox homeostasis, remodel the epigenetic landscape, and shape the tumor microenvironment (TME) [1, 2]. It is now clear that metabolic reprogramming is not a fixed property of cancer cells but a context‐dependent, evolutionarily plastic process that adapts to hypoxia, nutrient deprivation, immune pressure, and therapeutic challenge [3, 4].

These advances have been extensively documented in individual reviews, with most focusing on single pathways—glycolysis, lipid metabolism, or mitochondrial function in isolation—or on specific regulatory mechanisms such as HIF‐1α signaling or the Warburg effect [5, 6, 7, 8, 9, 10]. Other reviews have surveyed metabolic interactions within the TME or catalogued metabolism‐targeted therapeutic strategies without connecting these layers into a unified logic [11, 12]. Consequently, the field lacks an integrated framework that explains how distinct regulatory layers—transcriptional programs, posttranslational modifications, RNA‐based control, and microenvironmental crosstalk—converge on a limited set of metabolic hubs to coordinate plasticity, immune evasion, and therapy resistance across tumor types. Addressing this gap is particularly urgent given the growing recognition that metabolic compensation and heterogeneity are primary barriers to the clinical success of metabolism‐targeted therapies.

In this review, we organize tumor metabolic reprogramming into a functional‐module framework. Rather than treating glucose, lipid, cholesterol, and mitochondrial metabolism as independent pathways, we identify the regulatory hubs—most prominently the SREBP1/2–FASN–SCD lipogenic axis, the HIF‐1α–GLUT1–LDHA glycolytic axis, and the LDLR–LXR cholesterol homeostasis system—upon which diverse oncogenic signals converge. We further examine how posttranslational modifications, especially histone lactylation, and noncoding RNAs provide additional regulatory layers that fine‐tune these hubs in a context‐dependent manner, and how metabolic crosstalk between tumor cells and TME components—particularly tumor‐associated macrophages and cancer‐associated fibroblasts—amplifies these programs to drive immune evasion. By tracing the flow of regulation from oncogenic signals through convergence hubs to metabolic outputs and therapeutic phenotypes, this framework provides a coherent logic for understanding why tumors exhibit both remarkable plasticity and exploitable vulnerabilities.

The review opens by outlining the metabolic landscape of cancer, emphasizing the plasticity and spatiotemporal heterogeneity that distinguish tumor metabolism from normal tissue metabolism. It then examines the molecular mechanisms governing each metabolic module—from HIF‐1α‐driven glycolysis through SREBP‐centered lipid and cholesterol reprogramming to mitochondrial OXPHOS dependency—and how these modules are integrated through posttranslational modifications and microenvironmental crosstalk. Building on this mechanistic foundation, the discussion turns to the interconnections between pathways and the evaluation of therapeutic strategies, from single‐agent blockade through rational combination regimens to nanomedicine‐enabled metabolism–immunity reprogramming. The review closes by identifying the key challenges, limitations, and future directions that will determine whether tumor metabolism becomes a routinely actionable therapeutic axis.

## 2 The Metabolic Landscape of Cancer: Plasticity and Heterogeneity

**FIGURE 1:**
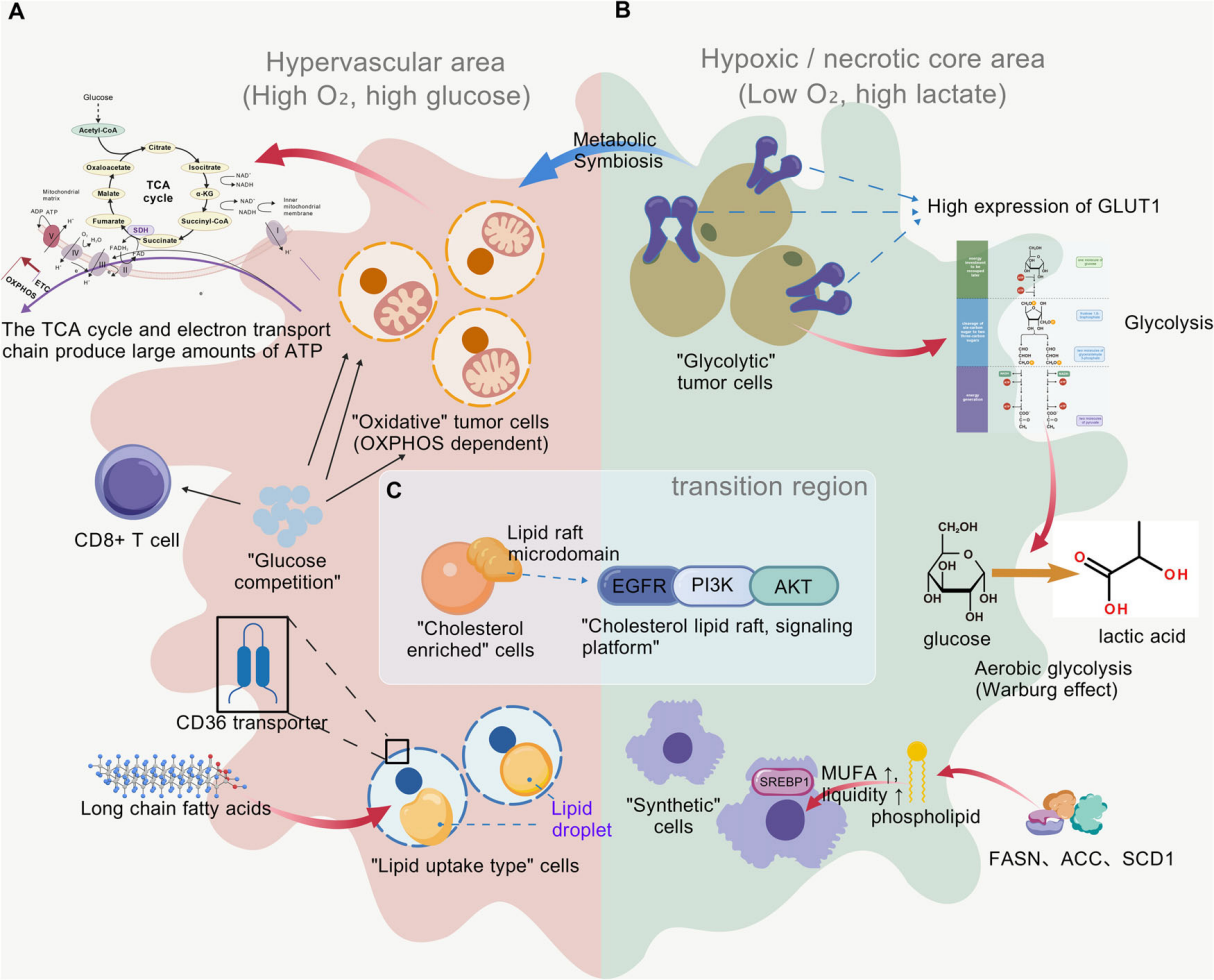
Metabolic landscape of tumors: spatial heterogeneity and plasticity. (A) Oxygenated/perfused region: Oxygen and glucose are relatively abundant, and tumor cells preferentially generate ATP through the tricarboxylic acid (TCA) cycle and mitochondrial oxidative phosphorylation (OXPHOS). In selected areas, lipid uptake and lipid metabolism provide additional fuel. Immune cells, such as CD8+ T cells, also compete for glucose, contributing to metabolic partitioning. (B) Hypoxic/necrotic core: This region is characterized by low oxygen tension, elevated lactate, enhanced glycolysis, and high GLUT1 expression. Lactate produced by glycolytic tumor cells is exported and exchanged with neighboring cells, supporting metabolic symbiosis between hypoxic core cells and surrounding populations. (C) Transition region: Cholesterol‐rich membranes and lipid‐raft domains are prominent in this compartment. EGFR–PI3K–AKT signaling promotes de novo lipogenesis, while FASN, ACC, SCD1, and SREBP1 support lipid synthesis, storage in lipid droplets, and membrane remodeling. This region illustrates the coupling of glycolysis with lipid metabolism during tumor proliferation and survival. Potential therapeutic targets highlighted in the figure include GLUT1, monocarboxylate transporters (MCTs), and key lipid‐metabolism regulators.

Beyond supplying ATP and biosynthetic precursors to sustain rapid proliferation, the tumor metabolic reprogramming network directly governs redox homeostasis, epigenetic regulation, and remodeling of the tumor immune microenvironment [13, 14]. Contemporary metabolic atlases reveal that cancer cells rarely depend on a single pathway; instead, they display pronounced metabolic hybridity and spatiotemporal heterogeneity, enabling adaptation to nutrient limitation, hypoxia, and therapeutic stress [15, 16] (Figure 1).

### 2.1 Glucose Metabolism: Glycolysis and Lactate Shuttling

Although aerobic glycolysis is a defining hallmark of malignant cells, its primary function is not ATP production per se, but the maintenance of high carbon flux to support nucleotide synthesis via the pentose phosphate pathway, amino acid biosynthesis (notably serine and glycine), and NADPH generation [17]. Recent work has redefined lactate from a metabolic waste product to a central currency of the tumor ecosystem. Glycolytic cells in hypoxic regions secrete lactate, which is taken up via MCT1 by oxidative tumor cells in oxygenated areas and converted to pyruvate to fuel the TCA cycle—a form of metabolic symbiosis [18]. In parallel, elevated lactate concentrations acidify the microenvironment to suppress T cell function and act as signaling molecules that induce histone lactylation, thereby directly regulating gene expression programs associated with tissue repair and immune evasion [19, 20] (Figure 1).

### 2.2 Mitochondria: Hubs of Bioenergetics and Macromolecular Biosynthesis

Contrary to earlier assumptions, functional mitochondria are indispensable for the vast majority of cancers [6, 21]. In particular, chemotherapy‐resistant cells, cancer stem cells (CSCs), and metastatic lesions frequently exhibit a marked dependence on mitochondrial OXPHOS—a phenomenon often described as OXPHOS addiction [22]. Continuous operation of the TCA cycle is sustained through glutamine‐driven anaplerosis, generating α‐ketoglutarate (α‐KG) and citrate, the latter being exported to the cytosol and converted into acetyl‐CoA to fuel lipid synthesis and histone acetylation [23, 24]. Moreover, TCA intermediates such as succinate, fumarate, and 2‐hydroxyglutarate (2‐HG) function as oncometabolites that inhibit α‐KG–dependent dioxygenases, thereby reshaping the epigenetic landscape and contributing to hypoxia‐inducible factor (HIF) stabilization [25]. Single‐cell and functional studies further reveal profound intratumoral heterogeneity in mitochondrial activity, with certain subpopulations even acquiring functional mitochondria via horizontal transfer to rescue impaired respiratory capacity [26, 27] (Figure 1).

### 2.3 Lipid Metabolism: Membrane Plasticity and Stress Tolerance

Reprogramming of lipid metabolism constitutes another central axis of tumor adaptation to hostile environments, encompassing both de novo lipogenesis and exogenous lipid uptake. The transcription factors SREBP1 and SREBP2 drive the upregulation of FASN, ACC, and SCD1, not only meeting the increased demand for membrane phospholipids during rapid proliferation, but also elevating the proportion of monounsaturated fatty acids (MUFAs) to maintain membrane fluidity and prevent lipid peroxidation–induced ferroptosis [5, 28, 29]. Under nutrient‐deprived conditions, tumor cells upregulate fatty acid transporters such as CD36, avidly scavenging extracellular lipids and storing them in lipid droplets as both energy reservoirs and buffers against metabolic stress [30, 31]. In addition, fatty acid oxidation (FAO) provides a substantial ATP supply for therapy‐resistant cells and is crucial for anchorage‐independent survival, enabling resistance to anoikis during metastatic dissemination [32, 33] (Figure 1).

### 2.4 Cholesterol and the Mevalonate Pathway: Structural Integrity and Survival Signaling

Cholesterol metabolism is tightly regulated through the mevalonate pathway, which not only produces cholesterol to maintain membrane rigidity and lipid raft integrity, but also generates key intermediates such as coenzyme Q10 and isoprenoid pyrophosphates. These metabolites are essential for GPX4 activity and for the membrane localization of small GTPases, including RAS and RHO family proteins [34, 35, 36, 37]. SREBP2 acts as a central regulator of this pathway and is frequently activated in cancer to support cell survival. Emerging evidence highlights a critical role for cholesterol‐derived metabolites, such as 27‐hydroxycholesterol, in modulating immune cell function; cholesterol‐rich tumor environments promote T cell exhaustion and establish potent immunosuppressive barriers [38, 39] (Figure 1).

### 2.5 Spatiotemporal Heterogeneity and Microenvironmental Crosstalk

The metabolic landscape of tumors is ultimately sculpted by the physical and chemical gradients of the tumor microenvironment (TME). Irregular vascularization generates gradients of oxygen and nutrients that force cancer cells to dynamically switch between metabolic states, thereby reinforcing metabolic plasticity [40]. At the same time, intense metabolic competition occurs between tumor cells and stromal components, including fibroblasts and immune cells [41]. Excessive glucose consumption by tumor cells restricts effector T cell cytotoxicity, while tumor‐derived metabolic by‐products such as lactate, adenosine, and kynurenine further suppress antitumor immunity [42, 43]. These metabolic checkpoints are a major driver of immunotherapy resistance [44] (Figure 1).

Collectively, cancer metabolism has evolved from a glycolysis‐centric view into a multidimensional network integrating glucose, mitochondrial, lipid, and cholesterol metabolism, thereby generating profound plasticity and heterogeneity. This metabolic flexibility enables tumors to exploit diverse substrates—including lactate, fatty acids, and glutamine—to survive under fluctuating microenvironmental pressures. At the same time, selective dependencies on specific metabolic pathways or nodes create therapeutic vulnerabilities, offering opportunities to disrupt tumor progression and overcome treatment resistance.

## 3 Advances in Tumor Metabolic Pathways and Molecular Mechanisms

Building on the metabolic landscape outlined above, this section examines the molecular mechanisms by which tumor cells execute and fine‐tune metabolic reprogramming. The discussion opens with the HIF‐1α–GLUT1–LDHA axis as the central driver of glycolytic metabolism, followed by the multilayered signaling convergence on the SREBP–FASN lipogenic hub, the reprogramming of cholesterol homeostasis through the LDLR–SCARB1–LXR system, and the mitochondrial amplification that generates OXPHOS dependency in therapy‐resistant and metastatic subpopulations. The final portion addresses the metabolic crosstalk between tumor cells and TME components—notably CAFs and TAMs—that shapes immune evasion and therapeutic responsiveness. A unifying theme across these five layers is the convergence of diverse oncogenic signals—genetic alterations, nutrient stress, hypoxia, and therapeutic pressure—onto a limited set of regulatory hubs and bottleneck enzymes. Posttranslational modifications, particularly histone lactylation, and RNA‐based regulatory mechanisms further provide a fine‐tuning layer that modulates metabolic flux with spatial and temporal precision. Tracing these mechanisms from individual pathways to the microenvironment yields a structured view of how metabolic plasticity is molecularly encoded.

### 3.1 Glycolytic Metabolism in Tumors

#### 3.1.1 Regulation of HIF‐1α and GLUT

Hypoxia‐inducible factor 1α (HIF‐1α) is a crucial transcription factor that regulates cellular adaptation to hypoxic environments and drives metabolic remodeling in various tumors [7, 45]. In the TME, hypoxic conditions often lead to the stabilization and activation of HIF‐1α, promoting tumor cell survival and proliferation [46, 47, 48]. Studies have shown that HIF‐1α enhances the glycolytic capacity of tumor cells by directly regulating the expression of glucose transporters (GLUTs) and glycolysis‐related enzymes [7, 49, 50], satisfying the energy demands for rapid proliferation [51].

**FIGURE 2:**
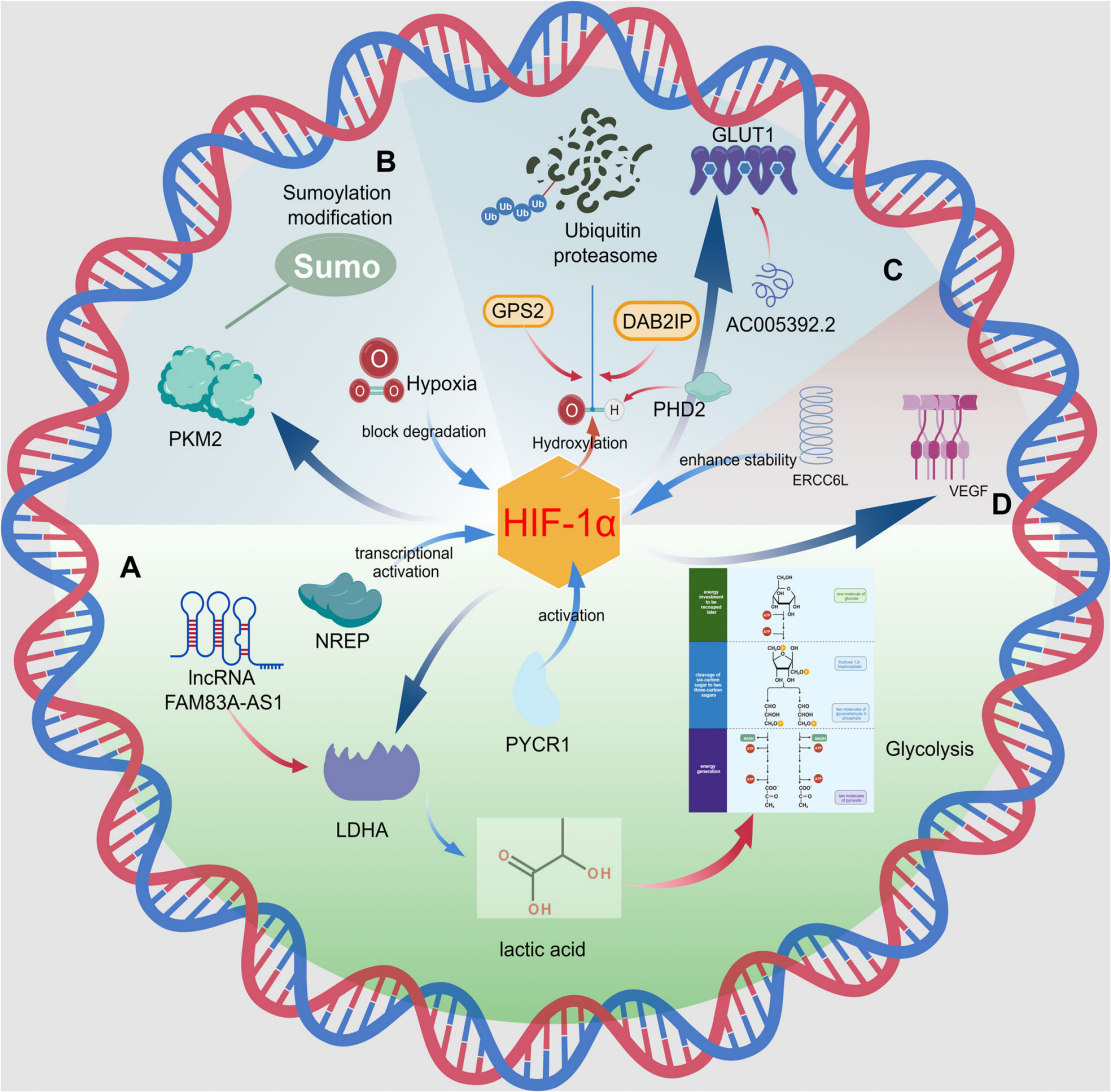
HIF‐1α regulates the central regulatory network of tumor glycolysis. (A) lncRNA FAM83A‐AS1, NREP, and PYCR1 enhance HIF‐1α‐driven glycolytic reprogramming, with LDHA contributing to lactate production and glycolytic flux. (B) Hypoxia stabilizes HIF‐1α by blocking its degradation, while PKM2‐associated SUMOylation and the ubiquitin–proteasome system further modulate HIF‐1α activity and turnover. (C) GPS2 and DAB2IP facilitate PHD2‐dependent hydroxylation and degradation of HIF‐1α, whereas AC005392.2 promotes GLUT1 stability and ERCC6L enhances HIF‐1α stability. (D) Activated HIF‐1α transcriptionally upregulates downstream programs involved in glucose uptake, glycolysis, lactate accumulation, and VEGF‐mediated adaptive signaling.

HIF‐1α activity and GLUT1 expression are governed by a multilayered regulatory network. At the transcriptional and posttranscriptional level, SOX2 drives expression of the lncRNA AC005392.2, which enhances GLUT1 stability by reducing H3K27me3 occupancy at its promoter [52]; HES1 activates the m^6^A reader IGF2BP2, stabilizing GLUT1 mRNA in colorectal cancer [53]; and NREP directly binds the HIF‐1α promoter to potentiate its transcriptional activity [54]. Noncoding RNAs add a further regulatory dimension: FAM83A‐AS1 sustains HIF‐1α and glycolytic signaling in lung adenocarcinoma [55]. Negative regulation is exemplified by DAB2IP, which promotes HIF‐1α ubiquitination and degradation, thereby restraining the HIF‐1α/GLUT1 axis in breast cancer [56]. Two positive‐feedback circuits further consolidate glycolytic commitment: the NUSAP1–LDHA–lactate loop, in which lactate‐driven lysine lactylation of NUSAP1 amplifies its own expression [57], and the ERCC6L–HIF‐1α loop, wherein ERCC6L inhibits HIF‐1α hydroxylation to prevent its degradation [58]. Metabolic enzymes also feed into this network: PYCR1 links HIF‐1α signaling to glutamine metabolism in bladder cancer [59]. These examples underscore a general principle—HIF‐1α is not a linear switch but the hub of a densely interconnected regulatory circuit that integrates transcriptional, posttranscriptional, and metabolic inputs (Figure 2).

#### 3.1.2 Role of Posttranslational Modifications

The regulatory scope of histone lactylation has expanded rapidly beyond its initial characterization. A dedicated enzymatic machinery is now recognized: HBO1 functions as a lysine lactyltransferase with its E508 residue serving as a catalytic site for histone Kla modification [60], while ACSS2 acts as a lactyl‐CoA synthetase that partners with KAT2A to deposit lactyl marks at H3K14 and H3K18 at promoters of Wnt/β‐catenin, NF‐κB, and PD‐L1 target genes; EGFR–ERK signaling triggers ACSS2 phosphorylation and nuclear translocation, directly coupling growth factor signaling to the lactylation landscape [61, 62]. Conversely, SIRT1 functions as a delactylase that attenuates H3K18la levels in gastric cancer [63], indicating that lactylation is dynamically regulated rather than passively reflecting lactate abundance.

H3K18 lactylation has emerged as the most recurrently implicated modification across cancer types. In colorectal cancer, H3K18la drives RUBCNL/Pacer transcription to promote cisplatin resistance [64] and is linked to GDF15–ENO1‐mediated lactate production in colonic epithelium [65]. In NSCLC, H3K18la enhances PD‐L1 expression and MYC nuclear transport [66], while TOP2A amplifies this axis by upregulating LDHA [67]. In breast cancer, H3K18la activates PPARD–AKT signaling [68], and newly identified sites H4K79la and H4K91la form a positive‐feedback loop that sustains glycolytic flux [69]. In HCC, H3K18la enhances NFS1 transcription to suppress ferroptosis sensitivity [70]; in bladder cancer, it drives cisplatin resistance through RBM15‐dependent m^6^A stabilization of IGFBP3 [71]; and in gastric cancer, elevated H3K18la correlates with tumor progression [63]. Beyond H3K18, H3K9 lactylation drives LAMC2 transcription in ESCC [72], while in TNBC the combined lactylation signature of H3K9la, H3K14la, and H3K18la shapes immune–microenvironment interactions [73].

Two additional themes warrant emphasis. First, lactylation extends beyond histones: PRMT1 lactylation enhances vimentin arginine asymmetric dimethylation to promote cell migration under hypoxia [74], and PADI1/3‐mediated citrullination of PKM2 creates crosstalk between lactylation and citrullination circuits [75]. Second, lactate‐driven positive‐feedback loops operate across multiple tumor contexts: GPR37 activates the Hippo pathway to upregulate LDHA, elevating H3K18la and CXCL1/5 expression in colorectal cancer liver metastases [76]; the LDHA–H4K12la–immune gene axis sustains immune evasion in PDAC [77]; and in melanoma, lactate‐mediated lactylation promotes angiogenesis through the IL‐33/ST2 axis [78]. ALKBH3, itself induced by histone lactylation, demethylates SP100A m^1^A modifications to suppress tumor suppressor formation in ocular melanoma [79], illustrating how lactylation can propagate its effects through additional epitranscriptomic layers. CCNB2 further links the cell‐cycle machinery to the glycolytic–lactylation network by driving H3K18la‐dependent transcription of TTK and BUB1B [80]. These findings, together with the H4K12la–SLFN5 axis that promotes TNBC progression [77], underscore the pervasive role of lactylation in coordinating metabolic flux, epigenetic state, and malignant behavior.

Together, these findings reveal several principles that transcend individual modification events. First, H3K18 lactylation (H3K18la) represents the most recurrent histone modification across cancer types, with functional roles documented in colorectal, breast, gastric, hepatocellular, bladder, and non‐small‐cell lung cancers (Table 1). This cross‐tumor recurrence suggests that H3K18la functions as a pan‐cancer node through which elevated glycolytic flux is transduced into transcriptional programs governing proliferation, immune evasion, and drug resistance. Second, lactate‐driven positive‐feedback loops are a recurrent architectural motif: glycolytic enzymes such as LDHA and PKM2 generate lactate, which in turn drives histone lactylation that transcriptionally upregulates glycolytic machinery—establishing self‐reinforcing circuits that lock tumor cells into high‐glycolytic states (exemplified by the NUSAP1–LDHA–lactate and TOP2A–LDHA–H3K18la axes). Third, the functional consequences of lactylation converge on three phenotypic modules: metabolic enzyme regulation that sustains glycolytic flux, immune evasion through upregulation of PD‐L1, B7‐H3, and CXCL1/5, and therapy resistance via enhanced DNA repair and ferroptosis resistance. Fourth, lactylation does not operate in isolation but crosstalks extensively with other posttranslational modifications—most notably through the ACSS2–KAT2A acetyltransferase complex, which couples lactate‐derived acetyl‐CoA to histone acetylation, and through the PADI1/3‐mediated citrullination of PKM2, which further potentiates glycolysis. Finally, the identification of dedicated lactylation machinery—writers (HBO1, KAT2A), erasers (SIRT1), and readers (CBX3)—indicates that histone lactylation is not merely a passive reflection of lactate abundance but an enzymatically regulated signaling system. Collectively, these observations argue that targeting the lactylation apparatus, rather than individual downstream targets, may offer a more generalizable strategy for disrupting the glycolytic–epigenetic feedback circuits that sustain aggressive tumor phenotypes. The functional diversity and convergence revealed by these studies are summarized in Table 1.

**TABLE 1:** Role of posttranslational modifications in tumor metabolism and associated mechanisms.

Posttranslational modification	Mechanism	Biological effect	References
H4K12 lactylation	Lactylation induced by lactate; inhibits SLFN5 expression, modulating tumor signaling and survival	Promotes tumor growth and survival	[77]
CCNB2‐related pathway	CCNB2 works with H3K18la to drive glycolysis; involves TTK/BUB1B	Affects tumor cell proliferation and metastasis	[64]
HBO1	HBO1 acts as a lysine lactyltransferase; E508 is critical	Regulates histone Kla modification	[60]
IGF2BP2/Nrf2 mRNA stability	Lactate increases IGF2BP2 expression and stabilizes Nrf2 mRNA	Enhances resistance to ferroptosis	[81]
GPR37	Activates Hippo pathway to boost LDHA expression and glycolysis; upregulates CXCL1/CXCL5	Increases glycolysis and proinflammatory chemokines	[76]
PRMT1 lactylation	Lactylation enhances asymmetric dimethylation at R64 (and related effects)	Increases cell migration under hypoxia	[74]
GDF15	Regulates ENO1 expression and increases lactate production; impacts histone modifications in CRC	Modulates tumor metabolism and epigenetic state	[65]
H3K18 lactylation	Regulates RUBCNL/Pacer transcription; promotes cisplatin resistance	Promotes drug resistance/tumor progression	[64]
H3K18 lactylation in Gastric cancer	Higher lactylation correlates with progression; SIRT1 lowers lactylation	Tumor progression	[63]
H3K18 lactylation in HCC	H3K18la increases NFS1 transcription; reduces ferroptosis sensitivity	Promotes hepatocellular carcinoma survival	[70]
H3K18 lactylation in NSCLC	Enhances PD‐L1 expression and MYC nuclear transport	Promotes tumor progression/immune evasion	[66]
H3K18 lactylation in Bladder cancer	RBM15 stabilizes IGFBP3 and affects DNA repair; associated with resistance	Drug resistance/progression	[71]
H3K18 lactylation in breast cancer	H3K18la increases PPARD expression and activates AKT signaling	Promotes tumor progression	[68]
H3K9/H3K14/H3K18 lactylation in TNBC	Lactylation features contribute to malignant transformation	Aids tumor malignancy in TNBC	[73]
Melanoma lactylation (IL‐33/ST2 axis)	Lactylation promotes angiogenesis via IL‐33/ST2	Promotes tumor growth and angiogenesis	[78]
ALKBH3 expression and SP100A m1A demethylation	Inhibits the formation of tumor suppressors	Promotes the malignant transformation	[79]
CHD4‐regulated PADI1/3 and PKM2 citrullination	CHD4 controls PADI1/3; PKM2 citrullination enhances glycolysis	Promotes glycolysis	[75]
H3K9 lactylation in ESCC	Regulates genes including LAMC2	Promotes esophageal squamous cell carcinoma progression	[72]
TOP2A regulation	LDHA and histone lactylation–driven glycolysis; promotes NSCLC proliferation and migration	Promotes tumor growth and spread	[67]
SOX9 lactylation	Lactylation of SOX9 promotes proliferation and metastasis	Promotes tumor growth and metastasis	[82]
PDIA3P1 in esophageal cancer	Increases glycolysis; upregulates GLUT1; inhibits HK2 ubiquitin degradation; enhances H4K8 lactylation; boosts BMP7 transcription	Promotes cancer progression	[83]
H4K79 / H4K91 lactylation	Positive feedback loop on glycolysis via lactylation	Promotes tumor progression	[69]
PYCR1 upregulation	Increases glycolysis and lactate production; raises H3K18la	Promotes proliferation, migration, invasion	[59]
H4K12 lactylation–immune axis	Lactylation axis promotes immune evasion	Immune evasion/tumor growth	[61]
ACSS2	ACSS2 forms lactyl‐CoA; interacts with KAT2A; EGFR‐ERK triggers H3K14/H3K18 lactylation; activates Wnt/β‐catenin, NF‐κB, PD‐L1	Promotes tumor growth and immune evasion	[62]

### 3.2 Tumor Lipid Metabolism

#### 3.2.1 Multilayered Signaling Convergence on the SREBP–FASN Axis

**FIGURE 3:**
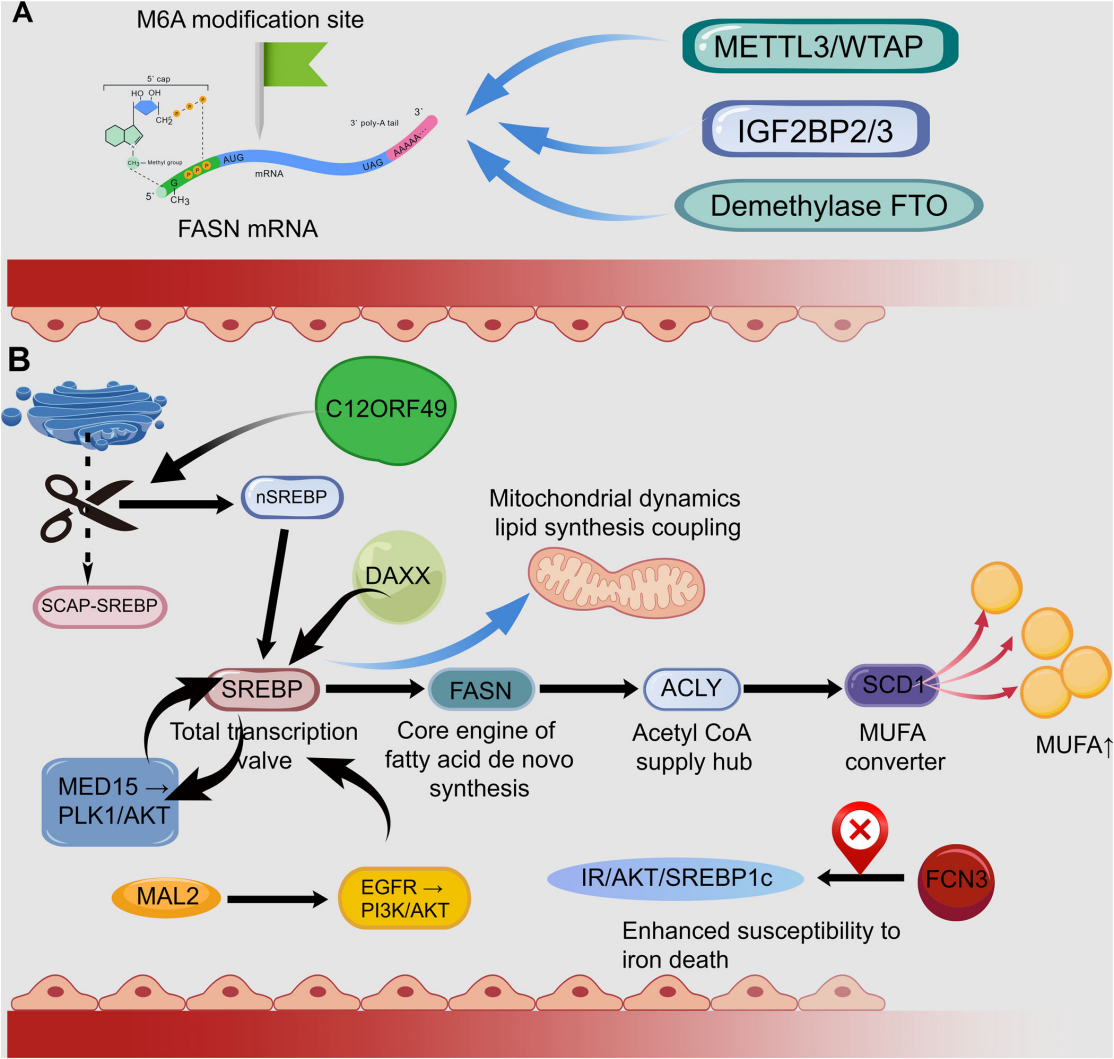
Multilayered convergence on the SREBP–FASN axis with RNA and epigenetic fine‑tuning of lipid metabolism in cancer. (A) Upstream convergence on the lipogenic axis: Multiple oncogenic and metabolic cues converge on the SREBP1/2–FASN–ACLY–SCD axis to enhance lipogenesis, lipid storage, ferroptosis resistance, and therapy adaptation. Representative regulators include DAXX, the MED15–PLK1–AKT–SREBP1/2 loop, C12ORF49, mitochondrial fission, MAL2, FCN3, PPARγ, and LR11. (B) RNA and epigenetic fine‐tuning: RNA‐based and epigenetic mechanisms fine‐tune lipogenic flux by modulating the expression, stability, trafficking, and activity of key lipogenic enzymes. These regulatory layers include m6A modifiers, AR‐driven alternative polyadenylation, circHIPK3, TENM3‐AS1, posttranslational controls, antilipogenic checkpoints, BCAT2‐mediated metabolic input, and chromatin modifications at lipogenic gene promoters.

Lipid metabolic reprogramming in cancer is characterized by diverse upstream activation routes but striking downstream convergence, with multiple signaling layers ultimately funneling into a limited set of lipogenic hubs centered on the SREBP1/2–FASN–ACLY–SCD axis. At the transcriptional level, SREBP1 and SREBP2 function as master regulators. DAXX directly enhances the transcriptional activity of SREBP1/2, coordinately amplifying fatty acid and cholesterol biosynthesis programs [84]. In clear cell renal cell carcinoma (ccRCC), MED15 activates PLK1–AKT signaling to establish a MED15–PLK1–AKT–SREBP1/2 positive feedback loop that sustains lipogenic output [85]. Similarly, C12ORF49 promotes SCAP–SREBP1 trafficking from the endoplasmic reticulum to the Golgi apparatus and subsequent proteolytic activation, thereby maintaining persistent nuclear SREBP1 activity across multiple solid tumors [86, 87] (Figure 3).

Organelle dynamics and receptor signaling further feed into this lipogenic axis. In intrahepatic cholangiocarcinoma, MAL2 impairs EGFR endocytic degradation, resulting in sustained PI3K–AKT–SREBP1 signaling and upregulation of FASN and SCD [88]. Conversely, in hepatocellular carcinoma (HCC), FCN3 suppresses the IR–AKT–SREBP1c axis, thereby reducing de novo lipogenesis and monounsaturated fatty acid (MUFA) production, and markedly increasing sensitivity to ferroptosis [89, 90] (Figure 3). More broadly, receptor‑ and transcription factor–driven programs involving PPARγ and LRP11 directly induce ACLY, FASN, SCD, and ACSL4 across breast, ovarian and other solid tumors, driving lipid droplet accumulation and neutral lipid loading that fuel acquired drug resistance and aggressive phenotypes by supplying both energy and membrane lipids [91, 92] (Figure 3).

#### 3.2.2 RNA and Epigenetic Fine‑Tuning of Lipid Metabolic Flux

Downstream of the SREBP–FASN backbone, RNA epigenetics, noncoding RNAs and posttranslational modifications constitute a multilayered fine‑tuning system that modulates lipogenic output with high precision. In ccRCC, the m^6^A demethylase FTO removes methylation marks from OGDHL mRNA, preventing TFAP2A ubiquitination and degradation, thereby transcriptionally activating FASN and amplifying both lipogenesis and ERK signaling [93]. In pancreatic neuroendocrine tumors, FTO demethylates APOE mRNA in an IGF2BP2‑dependent manner, stabilizing APOE, which in turn inhibits FASN ubiquitination and degradation, further enhancing FASN‑driven lipogenesis and PI3K–AKT–mTOR activation [94] (Figure 3).

The m^6^A writer–reader axis also directly targets FASN and SCD in multiple tumor types. In colorectal cancer, the METTL3–IGF2BP2/3 complex increases the m^6^A modification and stability of the lncRNA POU6F2‑AS1, enabling recruitment of YBX1 to the FASN promoter [95]. In cervical cancer and NSCLC brain metastases, IGF2BP3 stabilizes FASN mRNA and cooperates with METTL14 to recognize m^6^A sites on SCD mRNA, leading to coordinated upregulation of both enzymes [96, 97]. Beyond m^6^A, androgen receptor–driven alternative polyadenylation shortens the 3′ untranslated regions of FASN, ACSL3 and related genes in prostate cancer, allowing them to evade miRNA‑mediated repression [98] (Figure 3).

Non‑coding RNAs and post‑translational modifications further provide multiple regulatory entry points for key lipogenic enzymes. In esophageal squamous cell carcinoma, circHIPK3 acts as a competing endogenous RNA that sequesters miR‑637, relieving repression of FASN [99]. In gastric cancer, TENM3‑AS1 stabilizes hnRNPK and promotes its association with FASN mRNA, prolonging transcript half‑life [100]. At the protein level, the E3 ligase Riplet targets FASN for ubiquitination, and its loss leads to FASN stabilization [101]. In colorectal cancer and HCC models, FUT2‑mediated glycosylation of mSREBP‑1 and YAP1 cooperatively sustains high expression of FASN, ACC1 and SCD [102]. ZDHHC6‑dependent S‑palmitoylation of PPARγ prevents its lysosomal degradation, promotes nuclear translocation, and induces ACLY, ACC, FASN, and SCD1 expression [103]. By contrast, in renal cancer, the KAT2B–HDAC5 acetylation axis suppresses FASN promoter activity, representing a rare example of coordinated anti‑lipogenic and anti‑tumor regulation [104] (Figure 3).

Additional layers further integrate amino acid metabolism and chromatin state with lipid synthesis. In breast cancer, BCAT2 increases acetyl‑CoA availability to activate p300 and enrich Ac‑H3 at FASN and ACLY promoters [105]. In PDAC, TET3 relieves transcriptional repression of SCD and ACSL3 [106], while in PDAC, enrichment of the H3K4me3Q5ser mark at the SCD promoter underscores a direct coupling between histone modification and lipogenic gene activation [107]. Collectively, these findings demonstrate that amino acid metabolism, chromatin regulation, and lipid synthesis form a tightly integrated network across tumor types.

Despite the apparent diversity of upstream metabolic, receptor‑mediated, RNA‑based, and epigenetic signals, they converge structurally and functionally on a limited set of bottleneck enzymes and core transcription factors, notably SREBP1/2, FASN, ACLY, and SCD. This cross‑cancer convergence provides a strong conceptual and therapeutic rationale for targeting a small number of lipogenic hubs to disrupt multiple oncogenic pathways simultaneously.

### 3.3 Reprogramming of Tumor Cholesterol Metabolism

#### 3.3.1 Multidimensional Signaling Perturbations Reshape Cholesterol Homeostasis

**FIGURE 4:**
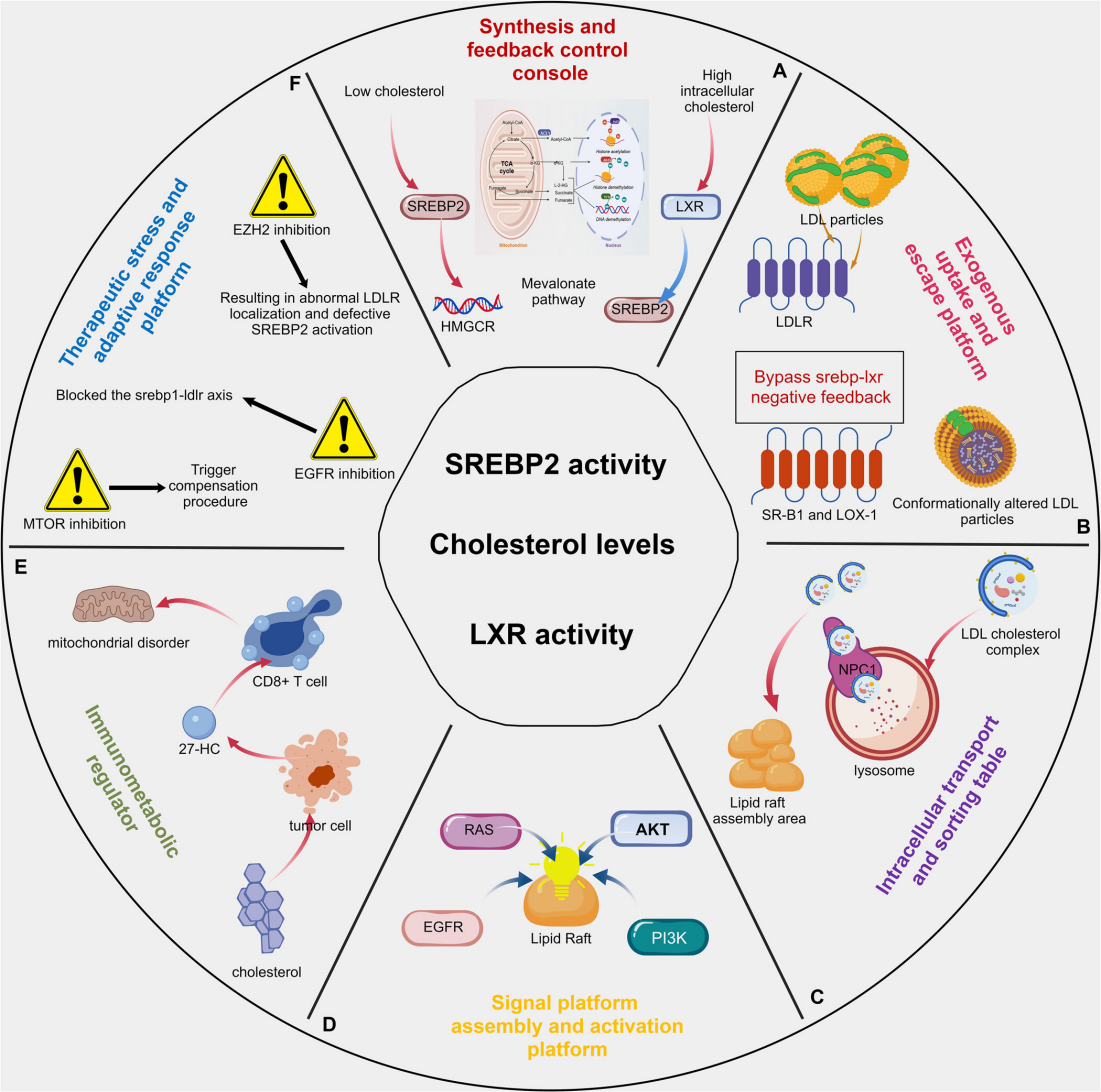
Multidimensional reprogramming and systemic adaptation of cholesterol metabolism in cancer. (A) LDL uptake and feedback escape: LDL receptor (LDLR)‐mediated cholesterol uptake increases intracellular cholesterol availability and may allow tumor cells to partially bypass canonical SREBP2‐ and LXR‐dependent feedback control. (B) Alternative lipoprotein uptake pathways: Conformationally altered LDL particles interact with scavenger receptors, including SR‐B1 and LOX‐1, thereby reshaping lipid homeostasis and evading conventional metabolic regulation. (C) Signaling platform for cholesterol trafficking: RAS–PI3K–AKT signaling promotes lipid‐raft assembly and activation, facilitating intracellular cholesterol transport and membrane‐associated signaling. (D) Cholesterol metabolism and antitumor immunity: Cholesterol imbalance and mitochondrial dysfunction impair CD8+ T‐cell activity, while 27‐hydroxycholesterol (27‐HC) further influences immune–tumor cell interactions within the TME. (E) Effects of targeted pathway inhibition: Inhibition of mTOR and EGFR disrupts the SREBP1–LDLR axis and triggers compensatory responses that reprogram lipid metabolism. (F) Feedback control of cholesterol homeostasis: Intracellular cholesterol levels dynamically regulate SREBP2 and LXR activity through feedback mechanisms involving the mevalonate pathway and HMG‐CoA reductase (HMGCR), thereby maintaining cholesterol balance.

As with fatty acid metabolism, aberrant cholesterol metabolism in cancer does not arise from linear amplification of a single pathway, but rather from the integrated effects of genetic alterations, nutrient availability, hormonal cues and therapeutic stress, leading to system‑wide reprogramming across cholesterol synthesis, uptake, trafficking and signaling. At the genetic level, the IDH1(R132H) mutation enhances production of 24(S)‑hydroxycholesterol (24‑OHC), activating liver X receptor (LXR) signaling to promote low‐density lipoprotein receptor (LDLR) degradation and cholesterol efflux, thereby establishing an oxysterol–LXR‑centered suppressive homeostatic program in glioma [108]. In contrast, tumors frequently bypass canonical feedback control through exogenous lipid supply. Diets enriched in saturated fatty acids induce conformational changes and aggregation of LDL particles, facilitating uptake through non‑canonical receptors such as LOX‑1 and LRP1, thereby escaping LXR–SREBP negative feedback and driving pathological lipid accumulation within the tumor microenvironment [109] (Figure 4).

This uptake‑dominant strategy is repeatedly observed across tumor types. In prostate cancer, persistent overexpression of SR‑B1 (SCARB1) maintains exogenous cholesterol influx to support steroidogenesis and androgen receptor signaling, a dependency that becomes particularly pronounced during castration‑resistant progression [110, 111]. Enhanced cholesterol uptake through LDLR and associated adaptor complexes similarly underlies resistance to lipid peroxidation and ferroptosis in ovarian cancer, esophageal squamous cell carcinoma and multiple myeloma [112, 113, 114] (Figure 4).

#### 3.3.2 Therapy‑ and Stress‑Induced Adaptations in Cholesterol Metabolism and Signaling Amplification

Beyond intrinsic supply routes, therapeutic interventions themselves actively reshape cholesterol dependencies and expose new metabolic vulnerabilities. In germinal center B‑cell–like diffuse large B‑cell lymphoma (GCB‑DLBCL), EZH2 inhibition disrupts LDLR subcellular localization and LDL uptake while limiting full activation of SREBP2, rendering tumor cells highly sensitive to cholesterol deprivation and inducing growth arrest and cell death [115] (Figure 4). By contrast, in multiple solid tumors, mTOR inhibition does not simply suppress lipid metabolism but instead induces adaptive mechanisms, including eIF3D–LRP6‑dependent alternative lipid uptake and LXRβ–NPC1‑mediated lysosomal cholesterol trafficking, which collectively preserve plasma membrane cholesterol and sustain IGF1R–AKT signaling, thereby promoting therapeutic resistance [116] (Figure 4).

This tight coupling between cholesterol homeostasis and oncogenic signaling correlates strongly with tumor aggressiveness and poor prognosis. Examples include basal‑like breast cancer, in which IGFBP6 downregulation drives a state of restricted uptake compensated by enhanced synthesis [117]; NSCLC, where EGFR inhibition blocks the SREBP1–LDLR axis, leading to lipid depletion and tumor cell death [118]; and pancreatic cancer, lung cancer and multiple myeloma, in which cholesterol‑enriched lipid rafts act as signaling platforms for PI3K–AKT, MAPK, and NF‑κB pathways, sustaining survival and metastatic potential [119, 120, 121]. Systemic metabolic status and microenvironmental cues are also deeply embedded within this network: elevated serum cholesterol increases the likelihood of vessel co‑option–type growth in colorectal cancer liver metastases [122], whereas oxysterols derived from lymphatic endothelial cells indirectly shape tumor progression through immune reprogramming [123] (Figure 4).

Despite extensive heterogeneity in the upstream regulation of cholesterol metabolism—spanning genetic, nutritional, hormonal and therapeutic dimensions—tumor cells ultimately achieve structural reprogramming of cholesterol homeostasis through dynamic modulation of a limited set of core nodes, including the SREBP–LDLR/SCARB1–LXR–NPC1 axis and lipid raft‐based signaling platforms. This cross‑tumor functional convergence provides a coherent and actionable framework for therapeutic targeting of cholesterol metabolism in cancer.

### 3.4 Mitochondrial Metabolism in Tumors

#### 3.4.1 Mitochondrial Amplification and Complex Activation

In ER+ breast cancer, endocrine therapy leaves residual tumor cells glycolytically depleted and energy‐starved. These cells survive and retain regenerative potential by increasing mitochondrial content and shifting dependence to OXPHOS. This response is echoed by the elevated mitochondrial biomass in ER+/HER2^−^ primary breast cancer that continues to proliferate after letrozole treatment [124]. In ccRCC, primary renal lesions exhibit low TCA cycle and electron transport chain activity (particularly complex I), whereas distal metastases reopen glucose‐ and acetate‐driven TCA cycles and respiration; modulating complex I activity is sufficient to confer or abolish metastatic capacity [125]. In bladder cancer, the mitochondrial translation activator TACO1 selectively enhances the translation of MTCO1, boosting complex IV activity [126]. In melanoma, weakening complex II or redirecting electron flow toward complex I reshapes succinate metabolism and histone methylation, upregulating MHC‐I and enhancing CD8+ T cell cytotoxicity independently of interferon [127].

#### 3.4.2 Tumor Metabolic Reprogramming Consolidates OXPHOS Dependency

Transcription factor‐driven OXPHOS addiction spans multiple tumor lineages. In AML and endometrial cancer, ERRα drives OXPHOS programs while shaping an immunosuppressive microenvironment [128, 129]. In ARMS and NSCLC, PAX3/7‐FOXO1 fusion proteins and the CIP2A–PKM2 axis reprogram enhancer networks and carbon flux distribution to enforce OXPHOS dependency [130, 131]. At the translational and assembly level, METTL1/WDR4‐mediated tRNA m^7^G modifications and SSR4 amplify mitochondrial respiration in gastric cancer by enhancing complex II and I/V assembly [132, 133]. In Kras‐G12D‐driven lung cancer, the AIF–CHCHD4 axis maintains complex I integrity, while Akt‐phosphorylated LonP1 clears damaged proteins from mitochondria under hypoxia and other stress conditions, ensuring efficient respiratory chain operation [134, 135]. Metabolic transporter and external inputs further consolidate OXPHOS dependency. In TNBC, over two‐thirds of ATP is derived from OXPHOS, with the glutamine transporter SLC6A14 further amplifying this dependency by promoting TCA flux and mitochondrial fusion [136, 137]. In HNSCC, Cav2+ neuronal fibers drive the transition from glycolytic to OXPHOS‐dominant metabolism through neuro‐tumor synapses, demonstrating that exogenous neuronal signals also shape mitochondrial dependence [138], indicating that exogenous neuronal signals also shape mitochondrial dependence.

These diverse mechanisms converge on a common phenotype: mitochondrial amplification coupled with OXPHOS dependency, driven by therapeutic pressure, metastatic bottlenecks, and microenvironmental cues. Rather than bluntly inhibiting the respiratory chain—an approach that would impair normal tissues—a more precise strategy would be context‐specific modulation of mitochondrial quantity, complex composition, and electron flow distribution, enabling selective targeting of tumor metabolism while preserving immune and normal tissue function.

### 3.5 Tumor Metabolism and the TME

#### 3.5.1 Role of Cancer‐Associated Fibroblasts and Tumor‐Associated Macrophages

**FIGURE 5:**
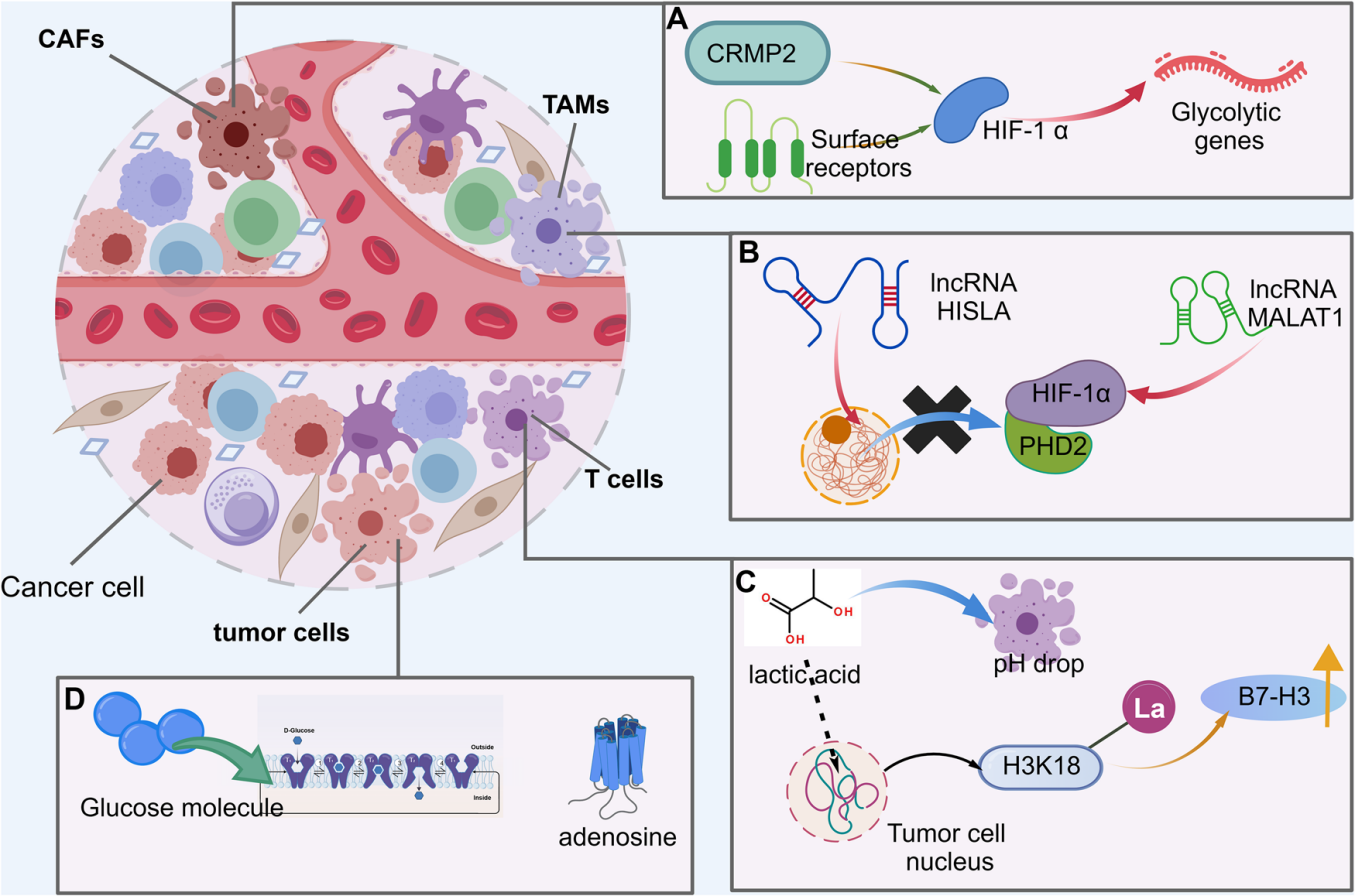
Metabolic crosstalk and immunometabolic regulation in the TME. (A) Cancer‐associated fibroblasts (CAFs) promote tumor glycolysis by secreting CRMP2, which activates HIF‐1α signaling in tumor cells. (B) Tumor‐associated macrophages (TAMs) reinforce the glycolytic phenotype by delivering exosomal lncRNAs, including HISLA and MALAT1, which stabilize or upregulate HIF‐1α signaling in tumor cells. (C) Tumor‐derived lactate suppresses T‐cell activity both by acidifying the microenvironment and by inducing histone H3K18 lactylation, which upregulates B7‐H3 expression in tumor cells. (D) Additional tumor metabolites, including adenosine and kynurenine, inhibit T‐cell receptor signaling and contribute to immune evasion.

Cancer‐associated fibroblasts (CAFs) are instrumental in shaping the TME, yet the mechanisms through which they drive ovarian cancer progression remain incompletely defined. CRMP2 secreted by CAFs activates the HIF‐1α glycolytic signaling pathway to promote ovarian cancer cell proliferation, migration, and invasion [139]. Tumor‐associated macrophages (TAMs) reprogram aerobic glycolysis in breast cancer cells by delivering long noncoding RNAs via extracellular vesicles. HISLA, a HIF‐1α‐stabilized lncRNA enriched in TAMs, inhibits the PHD2–HIF‐1α interaction, preventing HIF‐1α hydroxylation and degradation to drive aerobic glycolysis and treatment resistance. Tumor‐derived lactate further stimulates HISLA expression in macrophages, establishing a positive‐feedback loop between tumor cells and TAMs [140] (Figure 5).

In gastric cancer, M2‐TAM‐derived exosomes deliver lncRNA MALAT1 to tumor cells, where it facilitates aerobic glycolysis, proliferation, migration, and chemoresistance by inhibiting δ‐catenin ubiquitination and degradation while upregulating HIF‐1α. Dual inhibition of MALAT1 shows promise for improving chemotherapy sensitivity [141]. Lactate promotes B7‐H3 expression through histone lactylation: specifically, lactate‐driven H3K18 lactylation upregulates B7‐H3, which in turn suppresses tumor‐infiltrating CD8+ T cell abundance and function [142] (Figure 5).

#### 3.5.2 Metabolic Adaptation and Immune Evasion

In colorectal cancer, MPC1 and MPC2 elevate lactate levels, driving tumor cell proliferation, migration, and invasion while suppressing CD8+ T cell function through lactate‐induced histone lactylation; co‐overexpression of both transporters reverses these effects [143]. In glioblastoma stem cells (GSCs), lactate‐derived histone lactylation promotes immunosuppressive transcriptional programs and inhibits phagocytosis. Histone lactylation also enhances EP300 substrate specificity through CBX3 interaction, driving expression of immunosuppressive factors [144]. CAF‐secreted lactate elevates H3K18 lactylation in gastric cancer cells, upregulating ASPM, which facilitates NCAPG deubiquitination and enhances PD‐L1 expression [145] (Figure 5). In glioblastoma, monocyte‐derived macrophages (MDMs) outnumber microglia at later tumor stages and suppress T cell activity through glucose‐driven lactylation. Lactate‐driven histone lactylation is essential for IL‐10 expression in MDMs, sustaining their immunosuppressive activity and promoting cancer progression [146] (Figure 5).

Tumor cells enhance mitochondrial activity through metabolic adaptation in specific microenvironments, shaping an immunosuppressive environment. For example, in bladder cancer, the mitochondrial translation activator TACO1 selectively enhances the HIF‐1α‐dependent translation of MTCO1, improving the activity of complex IV and thereby influencing the metabolic state of cells [126]. Research in melanoma suggests that weakening complex II or biasing toward complex I to restore succinate metabolism can upregulate MHC‐I and enhance CD8+ T cell cytotoxic effects without relying on interferon [127]. In endometrial cancer, the estrogen‐related receptor ERRα drives OXPHOS programs while shaping an immunosuppressive microenvironment [128, 129]. Additionally, in triple‐negative breast cancer (TNBC), SLC6A14 amplifies dependence on OXPHOS by promoting TCA flux and mitochondrial fusion, resulting in over two‐thirds of ATP being derived from OXPHOS [136, 137] (Figure 5).

## 4 Interconnections Between Pathways

The metabolic modules described in the preceding sections—glycolysis, lipid synthesis, cholesterol metabolism, and mitochondrial OXPHOS—do not operate as parallel, independent tracks. Rather, they are extensively interconnected through at least four mechanistic principles: metabolite‐driven epigenetic coupling, enzyme moonlighting across compartments, signaling convergence on shared regulatory hubs, and metabolic competition within the TME. A growing body of recent literature, including several comprehensive reviews published in 2024–2025, has systematically documented these interconnections and elevated them from isolated observations to organizing principles of tumor biology [11, 12, 147]. Recognizing these principles is essential for understanding why single‐pathway therapeutic blockade so frequently fails, and why certain nodes—when targeted—produce effects that propagate across multiple metabolic modules.

### 4.1 Metabolite‐to‐Epigenome Crosstalk

**FIGURE 6:**
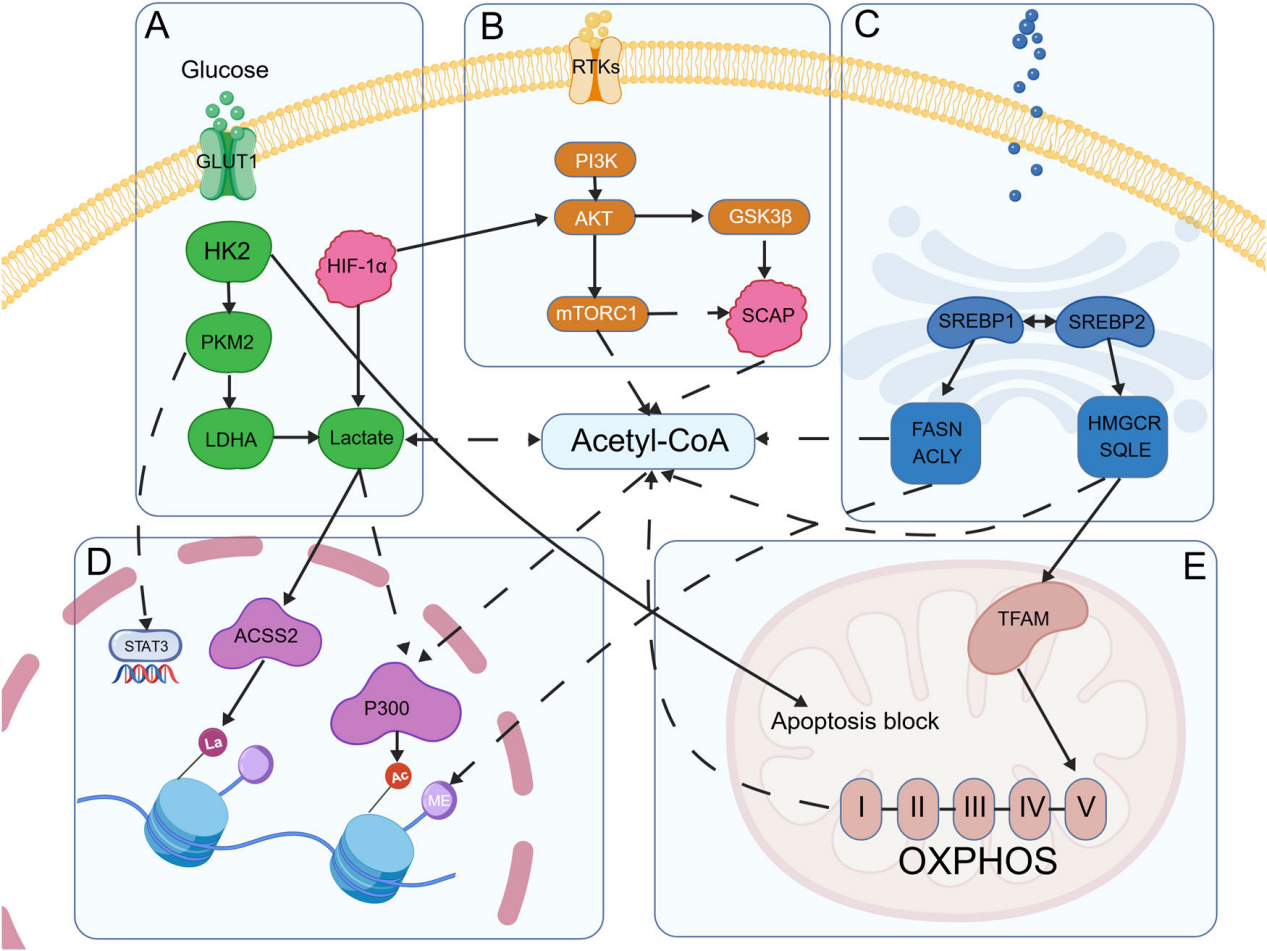
Interconnected metabolic circuits in cancer. (A) Glycolytic activation increases glucose uptake and lactate production, reinforcing HIF‐1α‐dependent metabolic programs. (B) RTK–PI3K–AKT–mTORC1 signaling converges on acetyl‐CoA production and SREBP activation through ACLY, SCAP and GSK3β. (C) SREBP1/2 coordinate fatty acid and cholesterol biosynthesis via targets including FASN, ACLY, HMGCR and SQLE. (D) Lactate and acetyl‐CoA couple metabolic flux to chromatin regulation through lactylation and acetylation mediated by ACSS2 and p300/CBP. (C) Moonlighting metabolic enzymes connect biosynthetic pathways to mitochondrial function and apoptosis control, exemplified by HK2‐mediated apoptosis blockade and SQLE–TFAM‐dependent regulation of OXPHOS. Solid and dashed arrows indicate direct regulatory events and intermodule metabolic coupling, respectively.

Metabolic intermediates function not merely as substrates and products but as direct regulators of the epigenetic machinery, creating bidirectional coupling between metabolic flux and gene expression. This concept, recently termed “metabolism–epigenetics intertwining,” has been comprehensively reviewed [148]. Acetyl‐CoA, the central two‐carbon currency of carbon metabolism, serves as the obligatory acetyl donor for histone acetyltransferases (HATs); ACLY and ACSS2, which generate acetyl‐CoA from citrate and acetate, respectively, are now recognized as critical nodes linking nutrient availability to chromatin state, with ACLY being directly phosphorylated and activated by AKT downstream of PI3K signaling [94, 149]. Lactate, the end product of glycolysis, drives histone lactylation—a modification whose regulatory scope has expanded dramatically since its initial description. Beyond H3K18la, the most recurrently implicated site across cancer types, global lactylome profiling has identified over 9000 nonhistone lactylation sites, including lactylation of adenylate kinase 2 (AK2) at K28 that directly promotes hepatocellular carcinoma metastasis [150]. The p300/CBP acetyltransferase complex has been identified as a key “writer” of lactylation marks, while ACSS2 functions as a lactyl‐CoA synthetase that partners with KAT2A to mediate histone lactylation at specific genomic loci, including promoters of Wnt/β‐catenin, NF‐κB, and PD‐L1 target genes [62]. These lactylation events create self‐reinforcing circuits: glycolytic activity generates lactate, lactate drives histone and protein lactylation, and the resulting transcriptional changes upregulate glycolytic enzymes (LDHA, HK2, GLUT1) and immune checkpoint molecules (PD‐L1, B7‐H3), further amplifying lactate production. In parallel, TCA cycle intermediates—α‐ketoglutarate, succinate, and fumarate—modulate the activity of α‐KG–dependent dioxygenases including TET DNA demethylases and Jumonji‐C domain histone demethylases, thereby coupling mitochondrial function to the DNA and histone methylation landscape [25]. Succinate and fumarate accumulation, as occurs in SDH‐ and FH‐mutant tumors, competitively inhibits these dioxygenases, producing a hypermethylated phenotype that silences tumor suppressors and promotes epithelial–mesenchymal transition [151]. These metabolite–epigenome loops create a form of metabolic memory: once a metabolic program is engaged, the resulting chromatin landscape tends to sustain it, contributing to the stability of drug‐tolerant persister states (Figure 6).

### 4.2 Enzyme Moonlighting across Compartments

Several metabolic enzymes possess dual—or in some cases, multiple—functions that physically bridge distinct metabolic compartments or link metabolism directly to signaling and transcriptional regulation. The human genome encodes approximately 1653 metabolic enzymes, vastly outnumbering the <800 protein kinases and phosphatases that constitute the canonical signaling machinery, suggesting a largely untapped reservoir of moonlighting potential [152]. Pyruvate kinase M2 (PKM2) exemplifies this principle: in its tetrameric form it catalyzes the final step of glycolysis in the cytoplasm, but upon oncogenic signaling—including growth factor stimulation and hypoxia—it dissociates into a dimer that translocates to the nucleus, where it binds tyrosine‐phosphorylated β‐catenin and functions as a transcriptional co‐activator for c‐Myc, upregulating glycolytic genes (LDHA, GLUT1) and EMT genes (Snail, Twist), while also directly phosphorylating STAT3 to promote transcription of genes involved in proliferation and fatty acid oxidation [153, 154]. Hexokinase 2 (HK2), beyond phosphorylating glucose, is tethered to the mitochondrial outer membrane via VDAC binding, where it physically prevents cytochrome c release and blocks intrinsic apoptosis—a function entirely independent of its catalytic activity [155]. HK2 also interacts with IκBα to activate NF‐κB, driving PD‐L1 transcription and directly coupling glycolytic rate to immune checkpoint expression [156]. Squalene epoxidase (SQLE), a cholesterol biosynthetic enzyme, aberrantly localizes to mitochondria in bladder cancer, where it binds the mitochondrial protease LONP1 and competitively inhibits degradation of the mitochondrial transcription factor TFAM, enhancing mitochondrial transcription and OXPHOS—a physical bridge between cholesterol metabolism and mitochondrial respiration [157]. These moonlighting functions carry an important therapeutic implication: inhibiting a metabolic enzyme may produce effects that extend unpredictably beyond its canonical pathway. Conversely, targeting the moonlighting function—for instance, blocking PKM2 nuclear translocation rather than its glycolytic activity—may achieve pathway selectivity that catalytic inhibitors cannot (Figure 6).

### 4.3 Signaling Convergence on Shared Metabolic Hubs

The same oncogenic signaling pathways simultaneously regulate multiple metabolic modules, providing a mechanism for coordinated reprogramming across glycolysis, lipogenesis, and cholesterol biosynthesis. The PI3K–AKT–mTORC1 axis is the most extensively characterized example of such convergence [149]. mTORC1 promotes the N‐glycosylation and proteolytic activation of SREBP1 and SREBP2 through SCAP, releasing them from Insig‐mediated ER retention for Golgi processing; AKT simultaneously inhibits GSK3β, preventing phosphorylation‐dependent ubiquitination and degradation of mature SREBPs, thereby sustaining their nuclear activity [35]. The net effect is the coordinated upregulation of: glycolytic transporters and enzymes (GLUT1, HK2), fatty acid synthesis enzymes (FASN, ACC, ACLY, SCD1), and the entire cholesterol biosynthesis and uptake machinery (HMGCR, SQLE, LDLR). This coordination explains why PI3K pathway activation alone is sufficient to drive simultaneous remodeling across multiple metabolic modules [149]. A recent study demonstrated that selective PI3Kδ inhibition in KRAS‐mutant colorectal cancer simultaneously suppresses AKT/mTOR/SREBP1‐mediated lipogenesis and triggers ferroptosis through the depletion of monounsaturated fatty acids, illustrating the therapeutic potential—and the pleiotropic consequences—of targeting this convergence node [158]. Beyond PI3K–AKT, KRAS mutations independently remodel the metabolic–epigenetic interface by upregulating GRAMD1A to enhance cholesterol transport while simultaneously increasing H3K9 lactylation to sustain glycolytic gene expression [159]. Tumor cell‐expressed PD‐L1, in addition to its immune checkpoint function, forms a complex with EGFR and ITGB4 that activates PI3K–AKT–mTOR–SREBP1c signaling, driving triglyceride and cholesterol accumulation—a function that positions PD‐L1 as a direct metabolic regulator in addition to an immune evasion molecule [160]. These examples underscore a general principle: oncogenic drivers achieve their metabolic effects not by activating pathways one at a time, but by engaging hub‐level regulators that coordinately reprogram entire metabolic sectors (Figure 6).

### 4.4 Metabolic Competition and Symbiosis in the TME

Inter‐pathway connections are not confined to the intracellular space. Within the TME, different cell types compete for shared substrates and exchange metabolites, creating intercellular connections that mirror the intracellular principles described above [11]. Tumor cells and CD8+ T cells compete for glucose, with excessive tumor glucose consumption—driven in part by the HIF‐1α–GLUT1 axis—directly limiting T cell glycolytic capacity and effector function [41]. Lactate secreted by glycolytic tumor cells is taken up via MCT1 by oxidative tumor subpopulations to fuel the TCA cycle, establishing a form of metabolic symbiosis between hypoxic and oxygenated tumor regions, while simultaneously suppressing CD8+ T cell and NK cell activity through both pH‐dependent mechanisms and lactylation‐dependent epigenetic reprogramming of immune cells [20]. In the lipid domain, a striking asymmetry has been documented: antitumor immune effectors (CD8+ T cells, NK cells, dendritic cells) depend on mitochondrial fatty acid oxidation for their effector and memory functions, yet are vulnerable to mitochondrial dysfunction in the nutrient‐poor, hypoxic, and lipid‐overloaded TME; conversely, tumor‐promoting immune populations—regulatory T cells, M2‐like TAMs, myeloid‐derived suppressor cells, and tumor‐associated neutrophils—gain a metabolic advantage by enhancing lipid uptake, synthesis, and mitochondrial utilization, driving immune suppression in similarly lipid‐rich environments [161]. Recent work has further demonstrated that the crosstalk between metabolism and cell death modalities—including ferroptosis (regulated by lipid peroxidation), cuproptosis (linked to mitochondrial metabolism), and necroptosis—adds an additional layer of interconnection, as metabolic states determine the threshold for different cell death pathways and thereby shape the immunogenic consequences of cell death within the TME [162]. These intercellular and inter‐pathway connections mean that metabolic therapy must be evaluated not only for its effect on the target cell type but also for the resulting perturbations to the broader metabolic ecosystem—a consideration elaborated in the therapeutic discussion that follows.

Collectively, these four principles—epigenetic coupling, enzyme moonlighting, signaling convergence, and intercellular competition—form a network of interconnections that explains both the robustness and the vulnerability of tumor metabolism. The robustness arises from redundancy: when one route is blocked, compensatory flux through interconnected pathways, or engagement of alternative moonlighting functions, can sustain essential metabolic functions. The vulnerability arises from convergence: diverse oncogenic and microenvironmental inputs ultimately funnel through a limited set of hubs—SREBP1/2, HIF‐1α, ACLY, p300/CBP, and ACSS2 among them—whose disruption propagates across multiple modules. This dual character—robust yet brittle—is the central tension that informs the design of metabolism‐targeted therapeutic strategies, as discussed in the following section.

## 5 Prospects of Metabolic Targeting in Antitumor Therapy: From Single‐Pathway Blocking to Nanoscales of Metabolism–Immunity Reprogramming

The molecular dependencies and convergence hubs identified in the preceding discussion provide the rationale for therapeutic intervention, but translating this knowledge into effective treatments requires navigating the very plasticity that defines tumor metabolism. The discussion begins with precision targeting of specific metabolic dependencies, in which single‐agent inhibitors exploit hardwired metabolic vulnerabilities in genetically or phenotypically defined tumor subsets. Recognizing that metabolic compensation frequently limits the durability of single‐agent responses, attention then turns to combination strategies designed to preempt compensatory rewiring—including dual metabolic blockade, chemo‐metabolic synergy, and metabolism–immunotherapy combinations that leverage immunometabolic remodeling to enhance antitumor immunity. The final portion examines how nanomedicine platforms are transforming metabolic therapy from systemic biochemical inhibition toward spatially and temporally controllable reprogramming of the tumor metabolic microenvironment, enabling co‐delivery, tumor‐selective activation, and simultaneous engagement of metabolic and immune targets. Across these three tiers, a common principle emerges: effective metabolic therapy must target not individual enzymes but the network‐level properties—convergence, compensation, and microenvironmental coupling—that define metabolic plasticity in cancer.

### 5.1 Precision Strikes on Specific Metabolic Dependencies

Despite the high plasticity of tumor metabolic networks, tumor cells exhibit “hardwired dependencies” on certain energy pathways in specific cancer types, stages of malignant transformation, or subpopulations, establishing a theoretical basis for precise metabolic targeting. Glycolytic targeting strategies cut off the carbon flux that sustains the Warburg effect. In precancerous lesions of gastric cancer and human high‐grade intraepithelial neoplasia (HGIN), metabolic defects are considered early events of malignant transformation. Proteomics and metabolomics analyses show that mitochondrial OXPHOS, particularly the function of complex I, is impaired. The traditional Chinese medicine LQWDD and its active component quercetin significantly inhibit malignant phenotypes by activating the AMPK signaling pathway to restore OXPHOS [163].

Conversely, in metastatic TNBC, OXPHOS serves as a survival pillar for the SOX2/OCT4+ stem cell‐like subpopulation. The copper chelator tetra thiomolybdate (TM) specifically blocks metastasis by depleting intracellular copper reserves and disrupting complex IV (cytochrome c oxidase), without affecting primary tumor growth [164] (Table 2). Similarly, streptomycin selectively inhibits mitochondrial‐encoded COX1 through its aldehyde structure, inducing iron‐dependent ROS‐mediated cell death and effectively eliminating resistant cancer stem cells in colorectal cancer [165]. In colon cancer, the combination of astragalus and curcumin (AC) affects tumor vascular normalization and antimetastatic capability by regulating hypoxia‐inducible factor‐1α (HIF‐1α) [166]. Selective serotonin reuptake inhibitors (SSRIs) exert antitumor effects in hepatocellular carcinoma, independent of their classic target SERT, by binding to GLUT1 and inhibiting HCC cell glycolytic metabolism [167]. Worenine inhibits cancer cell glycolysis and proliferation by negatively regulating HIF‐1α, thereby reversing the Warburg effect, where cancer cells depend on glycolysis rather than OXPHOS even in the presence of oxygen [168] (Table 2).

**TABLE 2:** Key metabolic targeting strategies and their tumor impact.

Key strategies	Mechanism	Impact	References
AMPK activation	Restores OXPHOS in gastric cancer	Inhibits malignant phenotypes	[163]
Copper chelation (TM)	Disrupts complex IV for stem cell‐like subpopulation	Blocks metastasis in TNBC	[164]
Streptomycin	Inhibits COX1	Induces ROS‐mediated cell death in CRC	[165]
Astragalus and curcumin	Regulates HIF‐1α	Affects vascular normalization in colon cancer	[166]
SSRIs	Binds GLUT1	Inhibits glycolytic metabolism in HCC	[167]
Worenine	Negatively regulates HIF‐1α	Reverses Warburg effect	[168]
CoQ0	Inhibits HIF‐1α	Prevents inflammation and EMT in HNSCC	[169]
GPS2	Degrades HIF‐1α	Inhibits aerobic glycolysis in breast cancer	[170]
Docetaxel	Meditates glycolysis via Smad3	Reduces lactate and HIF‐1α activity in prostate cancer	[171]
Leptosidin	Inhibits glycolysis	Induces apoptosis and reduces ATP in cancer cells	[172]
Corannulene derivatives	Targets GLUT1	Exhibits selective anticancer efficacy	[173]
FASN targeting (Orlistat)	Drives lipogenesis	Enhances sensitivity in CRC	[175]

CoQ0 inhibits the activation of the NLRP3 inflammasome by suppressing hypoxia‐induced HIF‐1α expression, lowering ROS generation in HNSCC cells, and preventing inflammation and cell migration. Additionally, CoQ0 upregulates epithelial markers like E‐cadherin and downregulates mesenchymal markers such as Twist and N‐cadherin, inhibiting epithelial–mesenchymal transition (EMT) and metastasis while diminishing glucose uptake, lactate accumulation, and the expression of various glycolytic enzymes, thereby weakening the Warburg effect [169] (Table 2).

GPS2 (G protein pathway suppressor 2) inhibits aerobic glycolysis in breast cancer by promoting RACK1‐mediated HIF‐1α degradation, thereby downregulating glycolytic gene expression [170] (Table 2).

Docetaxel suppresses prostate cancer cell proliferation and reduces lactate production by downregulating Smad3 phosphorylation, which in turn decreases HIF‐1α transcriptional activity. Exogenous Smad3 expression reverses docetaxel‐mediated suppression of HIF‐1α and its target genes [171]. Leptosidin induces apoptosis by inhibiting glycolysis and the SIRT1/GLUT1 signaling pathway, demonstrating cytotoxic effects and leading to significant reductions in intracellular ATP and lactate levels [172] (Table 2). Glycosylated conjugates of corannulene derivatives show potential as novel anticancer agents, particularly for their selective tumor‐targeting effectiveness. These conjugates exploit the Warburg effect unique to cancer cells to selectively uptake glucose transporters (GLUT1) and exhibit superior anticancer effects and safety profiles compared with control drugs in in vivo experiments [173].

In addition to mitochondrial targets, the single‐agent efficacy of targeting lipid synthesis pathways exhibits significant molecular feature dependency, providing a basis for stratifying patients. The sensitivity of fatty acid synthase (FASN) inhibitor orlistat is highly positively correlated with CSN6 expression levels: CSN6 drives lipogenesis by stabilizing FASN protein. In colorectal cancer patient xenograft models with high CSN6 expression, sensitivity to orlistat is significantly pronounced, while low expressers show essentially resistant traits [174, 175]. Moreover, in tumors driven by FUT2 overexpression, the enhanced proliferation, colony formation, and cytoskeletal remodeling dependent on palmitate can also be significantly reversed by FASN inhibitors [102]. These findings indicate that blocking a single metabolic pathway is not entirely ineffective; rather, it requires precise matching with specific dependencies between “metabolism and cellular states.”

### 5.2 Targeting Metabolic Plasticity and Synergistic Lethality

Single‐agent therapies are often limited by metabolic compensation; therefore, multitarget combination strategies aim to block compensatory pathways or exploit vulnerabilities induced by metabolic inhibition to enhance the efficacy of chemotherapy, immunotherapy, and radiotherapy [176, 177, 178, 179].

#### 5.2.1 Blocking Metabolic Compensation to Enhance Chemotherapy Sensitivity

Breast cancer cells establish a seesaw relationship between FASN‐mediated fatty acid synthesis and LDHA‐driven lactate production; simultaneous inhibition of both disrupts metabolic adaptability and reverses endocrine resistance [180]. In bladder cancer, the FASN inhibitor TVB‐3166 used in combination with gemcitabine eliminated chemotherapy‐induced tolerance to lipid peroxidation, significantly enhancing apoptosis [181]. A bispecific antibody targeting the hERG1–β1 integrin complex combined with statin drugs disrupts cholesterol‐rich lipid rafts to block the PI3K/AKT signaling pathway, significantly enhancing PDAC sensitivity to gemcitabine and oxaliplatin [120].

Patients with castration‐resistant prostate cancer (CRPC) exhibit elevated ^1^ ^8^F‐FDG PET SUVmax, which correlates with GLUT1 overexpression and poor prognosis. Mechanistically, GLUT1 is a direct transcriptional target gene of the androgen receptor (AR), promoting glucose uptake and glycolysis in tumor cells. Inhibiting GLUT1 not only significantly suppresses tumor growth and metabolism but also exerts a synergistic antitumor effect with AR inhibitors like enzalutamide, displaying notable efficacy in enzalutamide‐resistant models, positioning GLUT1 as an important potential therapeutic target in CRPC and resistant prostate cancer [182].

In TNBC, gemcitabine resistance frequently develops. Ganoderic acid D (GAD) reverses this resistance by inducing HIF‐1α degradation and consequently inhibiting glycolysis [183]. In tamoxifen‐resistant breast cancer, the G protein‐coupled estrogen receptor (GPER) stabilizes HIF‐1α by preventing its hydroxylation and ubiquitin‐mediated degradation, sustaining aerobic glycolysis and driving resistance [184].

#### 5.2.2 Reversing Immunosuppression through Metabolic Reprogramming

In Riplet‐deficient models, the combined use of FASN inhibitors and anti‐PD‐1 significantly restored CD8+ T cell function and prolonged survival [101]. In liver fluke infection‐induced tumor models, parasitic secretions stimulate lipid synthesis and enrich TAM (tumor‐associated macrophage)‐like macrophages; pharmacological inhibition of FASN can reverse this suppressive microenvironment and restore PD‐1 responses [185].

Furthermore, in 4T1 breast cancer models, the combination of orlistat and anti‐PD‐1 significantly increased CD8+ T cell infiltration. By upregulating 4‐HNE and COX‐2 while downregulating GPX4, this approach substantially enhanced tumor cell iron‐dependent apoptosis (ferroptosis) without noticeable toxicity [186]. Besides fatty acids, cholesterol metabolism also determines the outcome of immune surveillance. High expression of SQLE (squalene epoxidase) in tumors disrupts CD8+ T cell cholesterol homeostasis and function by secreting oxysterols (especially 27‐hydroxycholesterol); SQLE inhibitors like terbinafine can release this metabolic constraint and produce significant synergistic effects with anti‐PD‐1 treatment [187].

Golgi protein 73 (GP73) drives T cell antitumor function within the TME and is significantly upregulated during T cell activation and under hypoxic conditions. It regulates the mTOR–HIF‐1α signaling axis to maintain T cell glycolytic metabolism and enhance the effector functions of CD8+ cytotoxic T cells. T cells lacking GP73 show impaired HIF‐1α‐mediated glycolysis, resulting in decreased cytotoxic function and promoting tumor growth and metastasis, while overexpressing GP73 in T cells can restore metabolism and cytotoxicity and enhance the effectiveness of adoptive immunotherapy [188].

Under high‐glucose conditions, HK2 promotes PD‐L1 expression, suppressing CD8+ T cell function. The deletion of HK2 reduces the expression of CD274 mRNA in tumor cells, underscoring its significance in PD‐L1 expression. HK2 interacts with IκBα, activating the NF‐κB pathway to promote PD‐L1 transcription. Moreover, HK2 enhances CD133 expression in cancer stem cells (CSCs), thereby elevating the stemness of tumor cells [156]. The ApoA1 mimetic peptide L‐4F enhances CD8+ T cell antitumor capacity through HIF‐1α‐mediated glycolytic pathways [189]. Anti‐PD‐1 treatment may lead to upregulation of B7‐H3 expression, resulting in resistance to immunotherapy. Thus, combining lactate dehydrogenase inhibitors with anti‐PD‐1 treatment can significantly delay tumor progression [142].

#### 5.2.3 Metabolic Reprogramming Affects Radiotherapy Efficacy

Radiotherapy rapidly upregulates HMGCR, the rate‐limiting enzyme of cholesterol biosynthesis, in colorectal cancer cells, elevating intracellular cholesterol levels. This metabolic change causes STING protein retention in the endoplasmic reticulum, blocking interferon signaling mediated by the cGAS–STING pathway, thereby weakening CD8+ T cell‐mediated antitumor immunity. Blocking cholesterol synthesis can restore this innate immune pathway and overcome radiotherapy resistance [190].

Metabolic reprogramming of tumor‐associated neutrophils (TAN) in lung cancer also indicates that the glucose transporter GLUT1 is a key molecule maintaining TAN survival, promoting tumor functions and radiotherapy resistance. Compared with normal lung neutrophils, TAN exhibit higher GLUT1 expression and glycolytic activity, extending their lifespan in tumors and forming tumor‐promoting subpopulations characterized by high SiglecF expression. Neutrophil‐specific GLUT1 knockout accelerates TAN turnover and reduces tumor‐promoting subpopulations, suppressing tumor growth and enhancing radiotherapy effectiveness [191].

### 5.3 Nanomedicine‐Driven Metabolism–Immunity Reprogramming

Nanotechnology‐enabled approaches simultaneously optimize metabolic intervention and promote immune activation, opening new avenues for antitumor therapy.

#### 5.3.1 Nanotechnology Translates Metabolic Intervention Strategies Into Effective Treatments

Mitochondria are a primary target for nanocarrier‐based strategies. Acid‐sensitive PEG‐Dendron‐EPI@TPP‐LND nanoparticles use TPP‐mediated mitochondrial targeting to simultaneously block OXPHOS and compensatory glycolysis in TNBC cells, enhancing intracellular accumulation of chemotherapeutic agents [136]. Another peptide‐based in situ self‐assembling system forms nanofibers within cells, inducing mitochondrial autophagy through physical interference, resulting in lethal stress [192] (Table 3).

**TABLE 3:** Effects of nanotechnology‐driven metabolic interventions on tumor therapeutics.

Nanotechnology	Target	Effect	References
Mitochondrial nanocarrier	Blocks OXPHOS and compensatory glycolysis	Enhances chemotherapy efficacy	[136]
Self‐assembling peptide system	Induces autophagy	Promotes lethal stress in tumor cells	[192]
Black phosphorus nanosheets	Remodels metabolism	Reduces PD‐L1 expression	[193]
Dual blockade strategy	Sever mitochondrial hijacking in T cells	Protects energy and immunity	[194]
Enzymatic therapy with LNPs	Inhibits lipid synthesis	Prevents nuclear translocation of SREBP	[195]
Metformin + GOx	Suppresses glycolysis	Induces energy‐deficient state	[197]
Golgi‐customized Trojan Horse	Releases toxic agents	Enhances inhibition of glycolysis	[198]
Mitochondrial‐targeted RNAi	Induces mtDNA leakage	Activates immune response	[199]
D‐PPAR nanoparticles	Induces browning of white fat	Improves systemic environment	[200]
GF/Cu‐Pc@DA nanosystem	Blocks glycolysis and triggers cuproptosis	Enhances immunogenic cell death	[201]
PGM3 inhibition	Blocks hexosamine pathway/SREBP‐1 activation	Suppresses brain tumor growth	[196]

Exogenous black phosphorus nanosheets remodel metabolism by providing inorganic phosphorus and reducing PD‐L1 expression [193]. To thoroughly blockade metabolic plasticity, researchers have proposed “dual blockade” and “enzymatic therapy” strategies, where hyaluronic acid nanocarriers targeting CD44 simultaneously deliver nab‐paclitaxel (an OXPHOS inhibitor) and 3‐bromopyruvate (a glycolytic inhibitor), severing the mitochondrial hijacking pathways of T cells to achieve dual protection of energy and immunity [194].

In the context of enzymatic therapy, biomimetic peptides encapsulated in lipid nanoparticles (LNPs) can specifically block the PCK1–Insig interaction, thereby inhibiting the nuclear translocation of SREBP1/2 and lipid synthesis [195]. Interventions targeting the hexosamine pathway also block the N‐glycosylation activation signals of SREBP‐1 by inhibiting PGM3 [196] (Table 3).

Metformin (Met) selectively inhibits the glycolytic key enzyme HK2, thereby suppressing ATP production. Glucose oxidase (GOx) catalyzes glucose to generate toxic hydrogen peroxide, enhancing the energy‐deficient state of tumor cells. A self‐assembling metal‐organic framework ZIF‐8 has been developed as a co‐delivery system for Met and GOx, improving targeting efficacy of ZIF‐8 in the TME; additionally, RGD peptide modification further enhances ZIF‐8's targeting efficiency [197] (Table 3).

The Golgi apparatus is essential for protein transport, and Golgi phosphoprotein 3 (GOLPH3) is crucial for maintaining its function. The “Golgi‐customized Trojan horse” nanodiamond carrier effectively kills tumor cells by promoting the release of toxic molybdate via hydrogen peroxide and glutathione. At the same time, apigenin enhances its antitumor effects by inhibiting pyruvate kinase M2 (PKM2), and near‐infrared (NIR) irradiation can enhance the release of apigenin, further inhibiting the glycolytic process [198] (Table 3).

#### 5.3.2 Nanoplatforms Transform Metabolic Interventions Into Potent Immune Activators

Mitochondrial‐targeted RNAi nanoplatforms induce mtDNA leakage by silencing ATP6, activating the cGAS‐STING pathway, and polarizing macrophages to the M1 type, achieving an “energy kill + immune reversal” effect [199]. D‐PPAR nanoparticles targeting adipose tissue macrophages induce the “browning” of white fat by restoring lipid metabolism, improving the systemic environment [200] (Table 3).

Constructed to address hypoxic microenvironments, the GF/Cu‐Pc@DA nanosystem establishes a “dual‐engine switch.” This system first blocks glycolysis to limit lactate production and then triggers copper death (Cuproptosis) via copper‐phthalocyanine, improving oxygenation and enhancing immunogenic cell death induced by PDT [201] (Table 3). Collectively, these advances signal a transition in metabolic therapy: from single‐agent biochemical inhibition toward spatially and temporally controllable remodeling of the tumor metabolic microenvironment.

### 5.4 Clinical Translation Status and Strategic Prioritization

Despite the breadth of preclinical strategies reviewed above, inhibitors of mutant isocitrate dehydrogenase—ivosidenib for IDH1, enasidenib for IDH2—remain the only tumor metabolism–targeted drugs to have received FDA approval. This gap warrants a candid assessment of clinical successes and failures.

#### 5.4.1 Lessons From Failure

The glutaminase inhibitor telaglenastat (CB‐839) failed to improve progression‐free survival in the randomized Phase 2 CANTATA trial (NCT03428217; *n* = 444) in metastatic renal cell carcinoma (median PFS 9.2 vs. 9.3 months; HR 0.94; *p* = 0.65). The mitochondrial metabolism inhibitor devimistat (CPI‐613) showed no overall survival benefit in the Phase 3 AVENGER 500 trial (NCT03504423; *n* = 528) in metastatic pancreatic adenocarcinoma (median OS 11.10 vs. 11.73 months; HR 0.95; *p* = 0.66), with a higher rate of treatment‐related deaths (8.9% vs. 3.4%). First‐generation hexokinase 2 and lactate dehydrogenase A inhibitors encountered dose‐limiting toxicities due to the essential physiological roles of these enzymes in normal tissues. These failures converge on two obstacles: metabolic compensation through alternative pathways blunts single‐node blockade, and on‐target toxicity narrows the therapeutic window to a degree that preclinical models fail to capture.

#### 5.4.2 What Distinguishes the Successes

Ivosidenib achieved a complete remission rate of 32.8% in relapsed/refractory IDH1‐mutant AML (NCT02074839), and the Phase 3 AGILE trial (NCT03173248) demonstrated median overall survival of 24.0 versus 7.9 months with azacitidine alone (HR 0.44; *p* = 0.001) in newly diagnosed patients, with the CR rate tripled (47% vs. 15%). These agents succeed because they target a neomorphic enzymatic activity absent from normal cells and benefit from clear genetic biomarker‐driven patient selection—two features absent from most metabolism‐targeted strategies.

#### 5.4.3 Prioritization by Translational Readiness

Near‐term: agents targeting genetically defined metabolic vulnerabilities with existing companion diagnostics—mutant IDH inhibitors, SDH/FH‐deficient tumor strategies, and FASN inhibitors such as TVB‐2640 (currently in Phase I/II trials in prostate and breast cancer; NCT05743621, NCT03179904). Mid‐term: combination regimens pairing metabolic inhibitors with immunotherapy or chemotherapy, contingent on validated pharmacodynamic biomarkers of target engagement. Long‐term: nanomedicine‐enabled metabolic–immune reprogramming and computational model–guided adaptive therapy, which face barriers in manufacturing scalability, delivery efficiency, and regulatory frameworks. These challenges are examined in detail in the following section.

## 6 Challenges, Limitations, and Future Perspectives

The preceding sections have organized tumor metabolic reprogramming into functional modules—glucose, lipid, cholesterol, and mitochondrial metabolism—and have traced how these modules are regulated by signaling networks, posttranslational modifications, and microenvironmental crosstalk. Despite the conceptual coherence of this framework and the breadth of mechanistic evidence supporting it, translating these insights into clinical benefit remains a formidable challenge. Most metabolic inhibitors that showed promise in preclinical models have yielded disappointing results in clinical trials, and no metabolism‐targeted agent—with the notable exception of IDH inhibitors in acute myeloid leukemia—has achieved broad regulatory approval as a cancer therapeutic [202]. The following subsections critically examine the biological and methodological obstacles that account for this gap, identify limitations in the current evidence base, and outline priorities for moving the field toward clinically actionable metabolic interventions.

### 6.1 Key Challenges

Metabolic plasticity as a barrier to single‐agent therapy. A central challenge identified throughout this review is that tumor cells rewire their metabolic networks in response to pharmacological pressure. When glycolysis is inhibited, tumors may upregulate fatty acid oxidation; when FASN is blocked, compensatory lactate production can sustain survival [178]. This seesaw behavior—where blockade of one pathway induces compensatory flux through another—is not a rare exception but a predictable outcome of the network architecture described in Sections 3 and 4. It means that single‐agent metabolic inhibitors, however potent against their primary target, are unlikely to produce durable responses as monotherapies. The clinical implication is that effective metabolic therapy will require either rationally designed combination regimens that simultaneously block compensatory routes, or strategies that target the regulatory hubs—such as SREBP1/2 or HIF‐1α—upon which multiple pathways converge.

Inter‐ and intra‐tumor metabolic heterogeneity. As discussed in Section 2.5, metabolic phenotypes vary dramatically not only between tumor types but also across regions of the same tumor and between primary and metastatic lesions. A glycolytic core may coexist with an OXPHOS‐dependent invasive front; a treatment‐naïve primary tumor may be glycolytic, while its drug‐resistant recurrence may rely on mitochondrial respiration [128, 129]. This spatiotemporal heterogeneity means that biopsy‐based metabolic profiling—even when feasible—captures only a snapshot that may not represent the metabolic state of the entire tumor or its clinically most relevant subpopulation. Until methods for real‐time, spatially resolved metabolic monitoring become available, the ability to match patients to metabolism‐targeted therapies will remain limited.

The gap between preclinical models and human tumor biology. The vast majority of studies summarized in this review employed cell lines cultured under supraphysiological glucose and oxygen conditions, xenograft models lacking an intact immune system, or genetically engineered mouse models that recapitulate only a fraction of human tumor heterogeneity. The metabolic environment of a human tumor—with its complex vasculature, nutrient gradients, immune infiltrates, and stromal interactions—cannot be adequately modeled in these reductionist systems. For instance, the observation that atorvastatin can promote rather than suppress hepatocellular carcinoma progression under high‐fatty‐acid conditions [3] highlights how systemic metabolic status, which is absent from standard xenograft models, can fundamentally alter therapeutic outcomes. Organoid co‐culture systems, patient‐derived xenografts in humanized mice, and ex vivo tumor slice cultures offer incremental improvements, but each carries its own limitations in throughput, cost, or fidelity to the human TME.

Lack of clinically validated metabolic biomarkers. Even when metabolic vulnerabilities are correctly identified in a population, there are currently no widely accepted biomarkers for prospectively identifying which individual patients' tumors are dependent on a given metabolic pathway. FDG‐PET provides a gross readout of glucose uptake, but does not resolve the specific metabolic dependencies—OXPHOS versus glycolysis, FASN‐driven versus uptake‐driven lipogenesis—that determine sensitivity to targeted metabolic agents. Circulating metabolites, tumor transcriptomic signatures, and noninvasive metabolic imaging with hyperpolarized substrates are under investigation, but none have reached the level of standardization required for routine clinical decision‐making.

### 6.2 Limitations of Current Evidence

Beyond the broad challenges outlined above, several specific limitations in the current literature deserve mention. First, the field has produced conflicting evidence on the relative importance of glycolysis versus OXPHOS across cancer types. While the classical Warburg model emphasizes glycolytic dominance, functional studies in chemotherapy‐resistant breast cancer, melanoma, and AML have demonstrated that OXPHOS dependency is equally prevalent and may be the more clinically relevant target in certain contexts [128, 131, 132]. A systematic effort to map which tumor types and treatment contexts are glycolytic‐dominated, OXPHOS‐dominated, or hybrid is currently lacking.

Second, the signaling‐to‐metabolism regulatory logic—particularly the role of posttranslational modifications reviewed in Section 3.1.2—has been characterized predominantly at the level of individual modification events. While recurrent themes emerge (H3K18 lactylation is repeatedly implicated across breast, gastric, colorectal, and lung cancers; SREBP‐centered lipogenic convergence is observed across multiple tumor lineages), the field has not yet established whether these represent generalizable regulatory principles or context‐specific correlations. The functional redundancy and cross‐regulation among different PTM types (lactylation, acetylation, citrullination, SUMOylation) further complicate efforts to define causal hierarchies.

Third, the metabolic crosstalk between tumor cells and immune cells—while clearly important, as documented in Section 3.5—remains incompletely understood at a quantitative level. Measurements of absolute metabolite concentrations, fluxes, and exchange rates within the TME are technically demanding and rarely performed. Consequently, many of the immunometabolic mechanisms described in the literature rest on indirect evidence (gene expression changes, pharmacological inhibition) rather than direct demonstration of metabolic competition or transfer.

### 6.3 Future Perspectives

Toward metabolism‐based patient stratification. The single most impactful advance would be the development of robust, clinically deployable metabolic classification systems. Just as genomic subtyping has transformed the treatment of breast cancer (ER/PR/HER2) and NSCLC (EGFR, ALK, KRAS), a metabolic taxonomy—based on multi‐omics integration of transcriptomic, metabolomic, and imaging data—could guide the selection of metabolism‐targeted therapies. The metabolic subtypes already identified in TNBC (MPS1, MPS2, MPS3) [203] provide a proof of concept, but these classifications must be prospectively validated and linked to differential therapeutic responses before they can influence clinical practice.

Targeting convergence hubs rather than individual enzymes. The structural convergence identified throughout this review—where diverse oncogenic signals funnel through a limited set of metabolic bottlenecks (SREBP–FASN–SCD, HIF‐1α–GLUT1–LDHA, the LDLR–SCARB1–LXR cholesterol axis)—argues for a therapeutic strategy centered on these hubs. Unlike single‐enzyme inhibitors, hub‐targeting approaches may be less susceptible to compensatory bypass. Transcription factor‐targeted strategies (e.g., SREBP or HIF‐1α inhibitors), PROTAC‐mediated degradation of lipogenic enzymes, and bispecific antibodies that disrupt metabolic signaling platforms (such as the hERG1–β1 integrin–lipid raft complex [124]) represent promising directions. However, the challenge of achieving tumor‐selective targeting of transcriptional regulators without disrupting their physiological functions in normal tissues must be addressed through improved delivery systems and context‐specific activation strategies.

Rational combination design guided by metabolic network logic. Given the inevitability of metabolic compensation, combination therapy should be the default framework for metabolic intervention, not an afterthought. The most promising combinations—supported by the evidence reviewed in Section 5.2—pair a metabolic inhibitor with either (i) an agent that blocks the most probable compensatory pathway (e.g., FASN inhibition + LDHA inhibition [178]), (ii) an immunotherapy backbone that benefits from the immunometabolic remodeling induced by metabolic intervention (e.g., FASN inhibitor + anti‐PD‐1 [105, 184]), or (iii) a standard‐of‐care chemotherapy or radiotherapy regimen whose efficacy is enhanced by disrupting metabolic resistance mechanisms [179, 189]. Nanomedicine platforms, with their ability to co‐deliver multiple agents and achieve tumor‐selective release, are well‐suited to implement these combination strategies [138, 200].

Dynamic metabolic monitoring. The development of noninvasive methods for real‐time metabolic assessment—including hyperpolarized ^1^ ^3^C MRI, deuterium metabolic imaging, and metabolomics‐based liquid biopsy—could fundamentally change the way metabolic therapy is administered. Rather than treating based on a static baseline profile, clinicians could monitor metabolic adaptation during treatment and adjust regimens accordingly. In research settings, stable‐isotope tracing in patients (e.g., ^1^ ^3^C‐glucose or ^1^ ^3^C‐glutamine infusions) can reveal pathway activities that are invisible to steady‐state metabolite measurements, providing a more direct readout of therapeutic target engagement.

Integrative computational frameworks. Finally, the complexity and context‐dependence of tumor metabolism argue for computational approaches that can integrate the multiple data layers discussed in this review—genomic alterations, transcriptomic profiles, metabolomic measurements, imaging data, and clinical outcomes—into predictive models. Metabolic network models (genome‐scale metabolic models, constraint‐based flux analysis) and machine‐learning approaches trained on multi‐omics datasets could, in principle, predict metabolic vulnerabilities for individual tumors and simulate responses to combination therapies before they are tested empirically. The accuracy of such models will depend critically on the quality and diversity of training data, which in turn requires systematic metabolomic profiling of clinically annotated tumor cohorts—an effort that is now technically feasible but remains to be implemented at scale.

## 7 Discussion

This review has organized the complexity of tumor metabolism into a functional‐module framework, in which glucose, lipid, cholesterol, and mitochondrial metabolism are not treated as isolated pathways but as interconnected layers converging on a limited set of regulatory hubs—most prominently the SREBP–FASN–SCD lipogenic axis, the HIF‐1α–GLUT1–LDHA glycolytic axis, and the LDLR–SCARB1–LXR cholesterol homeostasis system. Posttranslational modifications, particularly histone lactylation, and microenvironmental crosstalk with CAFs and TAMs further fine‐tune these hubs, generating the plasticity that enables tumors to survive under diverse stressors.

A central insight emerging from this synthesis is that metabolic convergence on bottleneck enzymes and transcription factors is a double‐edged sword: it creates therapeutic vulnerabilities that can be exploited, but also enables rapid compensatory rewiring when individual pathways are blocked. This tension—between the attractiveness of hub‐targeted therapy and the reality of network‐level compensation—defines the current state of metabolic oncology and underscores why most single‐agent metabolic inhibitors have not translated into clinical practice.

The challenges, limitations, and future directions examined in Section 6 highlight the priorities for closing this gap: validated metabolic biomarkers for patient stratification, combination regimens designed around network logic rather than empirical trial‐and‐error, and dynamic monitoring tools that capture real‐time metabolic adaptation. Addressing these priorities will determine whether tumor metabolism becomes a routinely actionable therapeutic axis rather than just a well‐described hallmark.

## Author Contributions

C.J.C.: Conceptualization, Writing – original draft, visualization, Writing – review and editing. Y.X.H.: Data curation, Writing – review and editing. X.C.H.: Investigation. J.S.: Formal analysis. Z.Y.: Funding acquisition, supervision, conceptualization, Writing – review and editing. J.L.W.: Supervision, Writing – review and editing. S.C.: Funding acquisition, supervision, formal analysis, Writing – review and editing. All authors have read and approved the final manuscript.

## Funding

This work was supported by the National Natural Science Foundation of China (grant no. 82272985 to Shuo Chen); Guangzhou Municipal Science and Technology Project (no. 2024A03J0897; 2025A03J3761 to Shuo Chen); Guangdong Natural Science Foundation (no. 2025A1515012402 to Yang Zhao).

## Ethics Statement

The authors have nothing to report.

## Conflicts of Interest

The authors declare no conflicts of interest.

## Data Availability

All data generated or analyzed during this review are included in this published article.
